# Comprehensive Analysis to Reveal Amino Acid Metabolism-Associated Genes as a Prognostic Index in Gastric Cancer

**DOI:** 10.1155/2023/3276319

**Published:** 2023-05-11

**Authors:** Gangjun Zhao, Mi Wu, Qiuwen Yan

**Affiliations:** ^1^Affiliated Xiaoshan Hospital, Hangzhou Normal University, Hangzhou, China; ^2^Ningbo Medical Center Lihuili Hospital, Ningbo, China; ^3^The Affiliated Lihuili Hospital, Ningbo University, Ningbo, China; ^4^Medical School of Ningbo University, Ningbo, China

## Abstract

**Background:**

Amino acid metabolism (AAM) is related to tumor growth, prognosis, and therapeutic response. Tumor cells use more amino acids with less synthetic energy than normal cells for rapid proliferation. However, the possible significance of AAM-related genes in the tumor microenvironment (TME) is poorly understood.

**Methods:**

Gastric cancer (GC) patients were classified into molecular subtypes by consensus clustering analysis using AAMs genes. AAM pattern, transcriptional patterns, prognosis, and TME in distinct molecular subtypes were systematically investigated. AAM gene score was built by least absolute shrinkage and selection operator (Lasso) regression.

**Results:**

The study revealed that copy number variation (CNV) changes were prevalent in selected AAM-related genes, and most of these genes exhibited a high frequency of CNV deletion. Three molecular subtypes (clusters A, B, and C) were developed based on 99 AAM genes, which cluster B had better prognosis outcome. We developed a scoring system (AAM score) based on 4 AAM gene expressions to measure the AAM patterns of each patient. Importantly, we constructed a survival probability prediction nomogram. The AAM score was substantially associated with the index of cancer stem cells and sensitivity to chemotherapy intervention.

**Conclusion:**

Overall, we detected prognostic AAM features in GC patients, which may help define TME characteristics and explore more effective treatment approaches.

## 1. Introduction

Globally, gastric cancer (GC) ranks among the deadliest gastrointestinal disorders, accounting for 5.7% of all cancer diagnoses and causing more than a million cases annually; increases among people under 40 years of age [1, 2]. The current treatment for GC is surgical resection followed by fluorouracil and platinum-based chemotherapy. Unfortunately, GC is characterized by mild initial symptoms, a high degree of heterogeneity, distinct molecular kinds, and a range of biological characteristics. Most patients with GC are diagnosed late due to clinical relapse, distant metastases, inadequate treatment, and a poor prognosis. Chemotherapy-targeted drugs and immunotherapy are frequently employed to increase the survival rates of these patients. However, substantial systemic toxicity and rapidly developing drug resistance significantly reduce treatment efficacy [3]. Recently, the characterization of novel tumor subtypes based on expression profiling has contributed to a better understanding of molecular features and tumor heterogeneity in GC, such as the four molecular subtypes identified by a comprehensive molecular evaluation: the Epstein-Barr virus-positive tumors, unsteady microsatellite tumors, genomically secure lesions, and chromosomally unstable growths [4]; the three subtypes to describe the molecular and genetic characteristics of gastric adenocarcinoma [5]; the two molecular subtypes of metastatic gastric adenocarcinoma [6]; and three subtypes based on the altered proteome [7]. However, these molecular subtypes still face significant challenges in distinguishing patient prognosis and guiding personalized gastric adenocarcinoma treatment regimens.

Tumor cell metabolism is a key pathway that drives cancer stem cell survival, tumor cell transformation, immune evasion, drug resistance, and disease recurrence. Targeting tumor cell metabolism can enhance treatment responses to drug-resistant cancers and mitigate treatment-related toxicity by reducing the need for genotoxic drugs. Therefore, targeting tumor cell metabolism is a popular form of cancer treatment, especially amino acid consumption therapy, which has been the focus of recent research [8]. The categories of amino acid metabolism KEGG components include proline and aromatic amino acid metabolism and branched and branched-chain amino acid metabolism. A combination of signaling pathways and transcription factors often changes amino acid metabolic pathways in tumor cells [9, 10]. Cancer cells rely on foreign amino acid supply and meet increasing demand by upregulating the expression of the amino acid transporter. Interfering with amino acid availability is the Achilles heel unique to cancer [11]. Amino acids are also critical elements for immunological cells. T cells can upregulate amino acid transporter expression during proliferation, differentiation, and immunological response, increasing amino acid absorption and improving immune function [12, 13].

Regarding drug resistance, amino acids support cancer cells against therapy by maintaining biosynthetic processes, maintaining redox homeostasis, regulating epigenetic modifications, and providing metabolic intermediates for energy production [14]. For example, leucine or branched-chain amino acid therapy increases cisplatin sensitivity in cancer cells by suppressing cisplatin- or bcat1-mediated autophagy and promoting mTOR signaling [15].

In this study, our aim was to investigate the characteristics of AAM-related genes in GC systematically and comprehensively. First, we used TCGA-STAD and GSE84337, which were obtained from the Cancer Genome Atlas (TCGA) database and GEO database, to analyze that the genome associated with amino acid metabolism (AAM) could divide GC into different subgroups. We then evaluated molecular signatures and infiltrative immune cell strength to identify AAM clusters. In addition, risk profiles based on four genes were confirmed as independent prognostic factors for gastric cancer, suggesting an association between amino acid metabolism-related genes and prognosis. Finally, we determined an AAM score that significantly predicted clinical outcomes and medication therapy effects in patients with gastric cancer. These findings might open up new avenues for GC study and customized therapy.

## 2. Materials and Methods

### 2.1. Data Collection and Collation

TCGA-STAD tool (https://portal.gdc.cancer.gov) was used to retrieve information on RNA expression, somatic mutations, copy number variation (CNV) files, and related GC clinicopathology., Moreover, GSE84337 from the GEO archive (https://www.ncbi.nlm.nih.gov/geo/) was used to obtain clinical parameters and normalized gene expression data. Two datasets were combined, and batch effects were eliminated by applying the “Combat” algorithm [16]. A total of 101 AAM genes were discovered in older research in addition to the amino acid and derivative metabolic process gene list in the Molecular Signatures Database (MSigDB) (https://www.gsea-msigdb.org/gsea/index.jsp) (Table S1). STRING analysis (https://string-db.org/) was utilized to illustrate interactions between AAM-correlating genes.

### 2.2. Differential Expression and Mutation Analysis of AAM Genes

Differential expression AAM genes were identified in TCGA-STAD dataset by the limma package in R software [17]. The landscape of AAM gene mutations was illustrated by the maltools package's waterfall graph, while changes in CNV placements of AAM genes on chromosomes were mapped by the RCircos program [18].

### 2.3. Consensus Clustering

The different AAM correlation modes were defined by the ConsensusClusterPlus package [19] and the K-means method. These steps have been performed 1000 times to guarantee the stability of the categories. Then, the clustering results were validated using principal component analysis (PCA) [20]. The clinical significance of the clusters was determined by evaluating molecular patterns, clinical variables, and patient outcomes. Additionally, GSVA enrichment analysis was performed in the heatmaps using the GSVA program to evaluate if the verified gene sets differed significantly across three clusters [21]. Additionally, a single-sample gene set enrichment analysis (ssGSEA) was applied to examine the differences in immune cell infiltration proportions between subgroups.

### 2.4. Differentially Expressed Genes (DEGs)

Package “limma” in R [17] was used to identify DEGs between different AAM molecular subtypes, with the criterion of |log2FC| > 0.585 and false discovery rate (FDR) < 0.05. Genes that intersect or do not intersect between subgroups were visualized using Venn diagrams.

### 2.5. Development of a Risk Signature Based on Clusters of AAM

AAM score was constructed to quantify amino acid metabolism in GC patients. Intersect genes were chosen based on DEGs expression data in various clusters of AAM across GC samples. The 65 intersect genes associated with prognosis were screened and analyzed by univariate Cox regression. Genes linked with AAM were scored using PCA through the following technique: AAM score = *Σ*(Expi × coefi). Then, TCGA-STAD and GSE84437 cohorts were used for further analysis. A nomogram was created from risk scores and clinical data using the rms program [22] to predict the overall survival (OS) of patients with GC at one, three, and five years. The stromal, immunological, and ESTIMATE scores were examined using the ESTIMATE algorithm [23] to determine the relationship between the risk score and the tumor immune microenvironment.

### 2.6. Chemotherapy Sensitivity Prediction

To explore differences in chemotherapy sensitivity between groups, we evaluated the highest half-maximal inhibitory concentration of chemotherapy drugs (IC50) by R-package “pRRophetic” [24].

### 2.7. Statistical Analysis

All statistical tests were performed using R software, version 4.2.0, and the relevant feature packages. Differences between different datasets were determined using the Chi-square test. Two groups were compared using the Wilcoxon test. The log-rank test was applied to determine the Kaplan-Meier (KM) survival analysis. *P* values below 0.05 were classified as statistically significant (^∗^*P* < 0.05; ^∗∗^*P* < 0.01; ^∗∗∗^*P* < 0.001).

## 3. Results

### 3.1. AAM Gene Expression Analysis and Mutation Analysis in STAD

**Figure 1:**
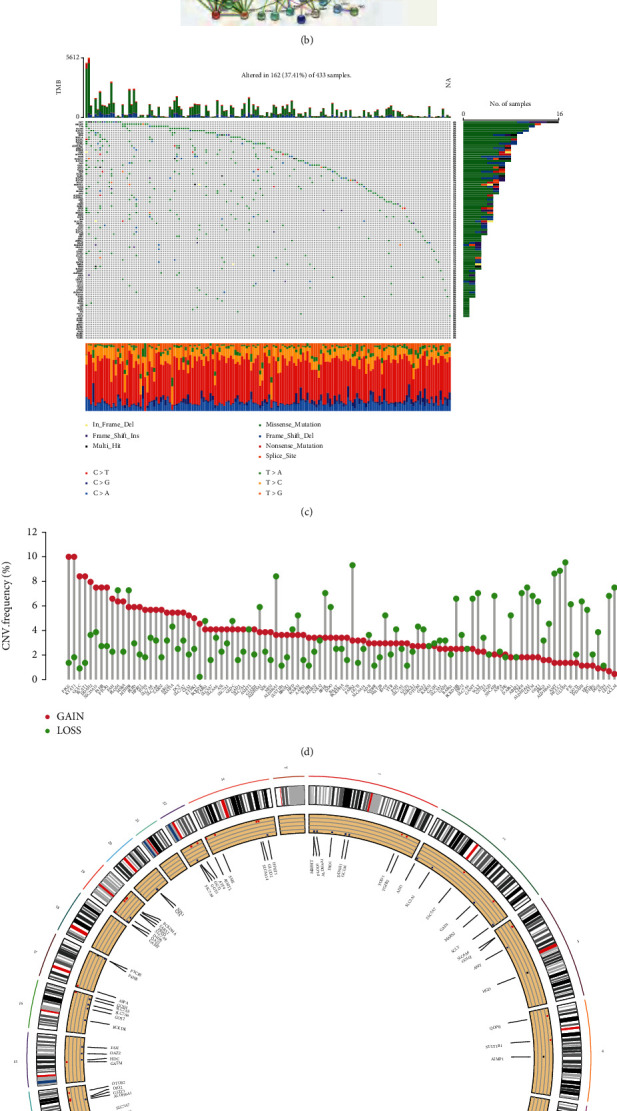
Genetic mutational landscape of AAMs in GC. (a) Expression distributions of DEGs between GC and normal tissues. (b) The PPI network acquired from the STRING database among the DEGs. (c) Genetic alteration on a query of *AAMs*. (d) Frequencies of CNV gain, loss, and non-CNV among AAMs. (e) Circus plots of chromosome distributions of AAMs. (^∗^*P* < 0.05; ^∗∗^*P* < 0.01; ^∗∗∗^*P* < 0.001).

We examined 101 AAM gene expressions in tumor and normal tissue samples using the TCGA-STAD dataset. 79 AAM genes were either up or downregulated in STAD (Figure 1(a)). At the protein level, the interactions between the 101 AAM gene proteins were analyzed using STRING and mapped the PPI network (Figure 1(b)) The incidence of somatic mutations and CNVs of AAM-related genes in GC patients was assessed. Only 163 of 433 samples contained mutations in AAM-related genes, with a mutation frequency of 37.41%, and the data implicated DCT as the gene with the highest mutation frequency (4%) (Figure 1(c)). Missense mutations are the most common type of gene alteration. We also discovered that at the CNV level, the focus was mostly on CNV loss. FPGS and KYAT1 have a broad rate of CNV gain (Figure 1(d)). Additionally, we discovered alterations in 95 AAM genes with chromosomal CNV characteristics (Figure 1(e)).

### 3.2. AAM Patterns in GC

**Figure 2:**
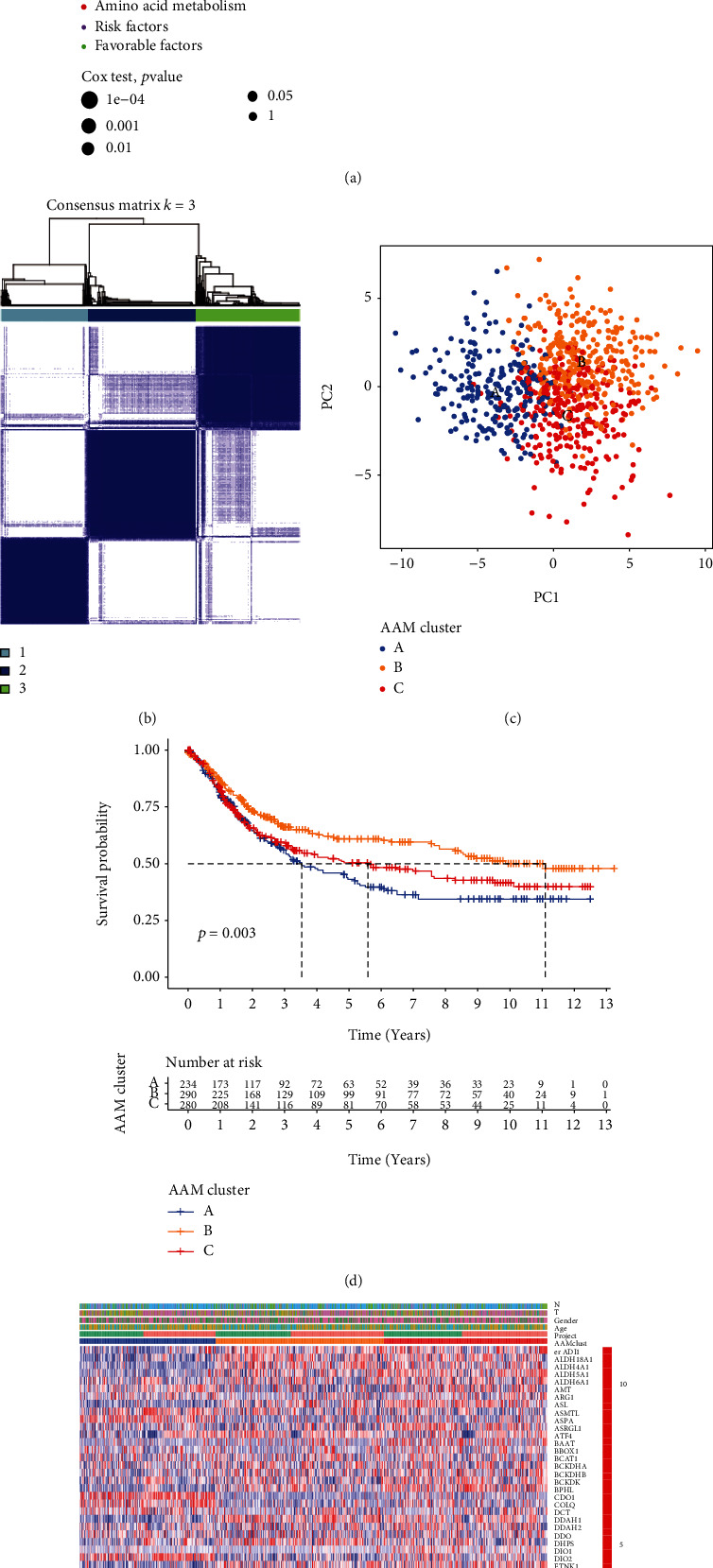
AAM subgroups and clinicopathological and biological characteristics of three distinct subtypes of samples divided by consistent clustering. (a) A network of correlations including AAMs in the TCGA cohort. (b) Consensus matrix heatmap defining three clusters and their correlation area. (c) PCA analysis indicating an obvious difference in transcriptomes among the three subgroups. (d) Univariate analysis showing three distinct subtypes correlated with OS. (e) Differences in clinicopathologic characteristics and expression levels of AAMs among the three distinct subgroups.

We combined TCGA-STAD and GSE84337 transcriptome data and retrieved the mRNA expression data for 101 AAM genes; Table S2 lists the OS statistics and clinical details for these subjects. Table S2 provides information on these subjects. Ninety-one genes in GC have prognostic scores determined using univariate Cox regression analysis and KM analysis (Table S3). The regulator network illustrated the entire landscape of 91 gene connections, regulator relationships, and their prognosis for GC patients (Figure 2(a)). The above data showed that AAM might significantly characterize TME cell infiltration within specific tumors. Patients were categorized using the ConsensusClusterPlus R package based on the expression of the 91 AAM genes. The unsupervised clustering technique divided the data into three distinct groups: 234 cases of model A, 291 cases of model B, and 282 cases of model C (Figure 2(b)). PCA analysis also showed a good distribution among groups (Figure 2(c)). The cluster B model provided a significant health benefit (Figure 2(d)). Moreover, Figure 2(e) shows that both cluster's genomic expression and clinic pathological factors were compared, revealing a significant difference in AAM gene expression and clinical characteristics.

### 3.3. Different Clusters' TME Characteristics

**Figure 3:**
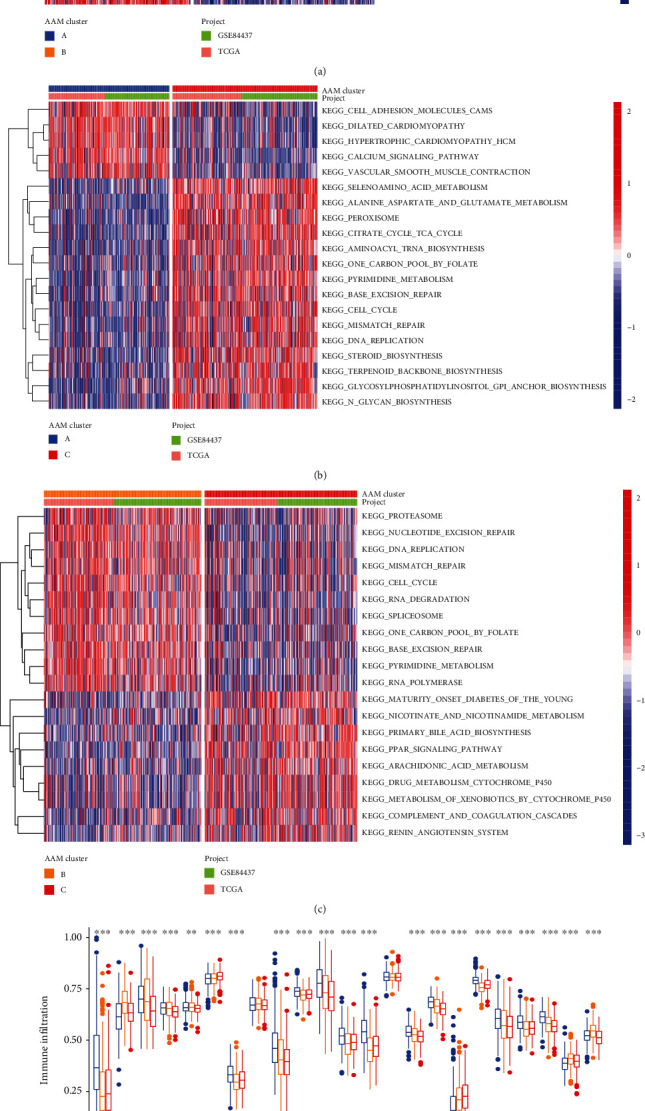
Different clusters' TME characteristics. (a–c) GSVA of biological pathways among three distinct subgroups. (d) The abundance of each TME infiltrating cell in three AAM clusters.

GSVA enrichment analysis revealed that cluster A was greatly elevated in cardiovascular pathways, including vascular smooth muscle contraction, dilated cardiomyopathy, and hypertrophic cardiomyopathy (Figure 3(a)). Cluster C described the enrichment mechanisms for metabolism. These included the alanine, aspartate, glutamate, arachidonic acid metabolisms, toxic substances metabolism by cytochrome P450, and nicotinate and nicotinamide metabolisms (Figure 3(c)). Cluster B was significantly associated with nucleic acid anabolism (Figure 3(b)).

Furthermore, we analyzed the immune cell infiltration of three clusters using the ssGSEA technique. Cluster A contained a significantly high number of innate immune cells, such as activated B cells, activated CD4 T cells, activated CD8 T cells, activated dendritic cells (DC), eosinophils, immature B cells, immature DC, MDSCs, macrophages, mast cells, natural killer T (NKT) cells, natural killer (NK) cells, plasmacytoid DC, regulatory T cells, and T helper cells (Figure 3(d)).

### 3.4. Generation of AAM-Related Gene Signatures

**Figure 4:**
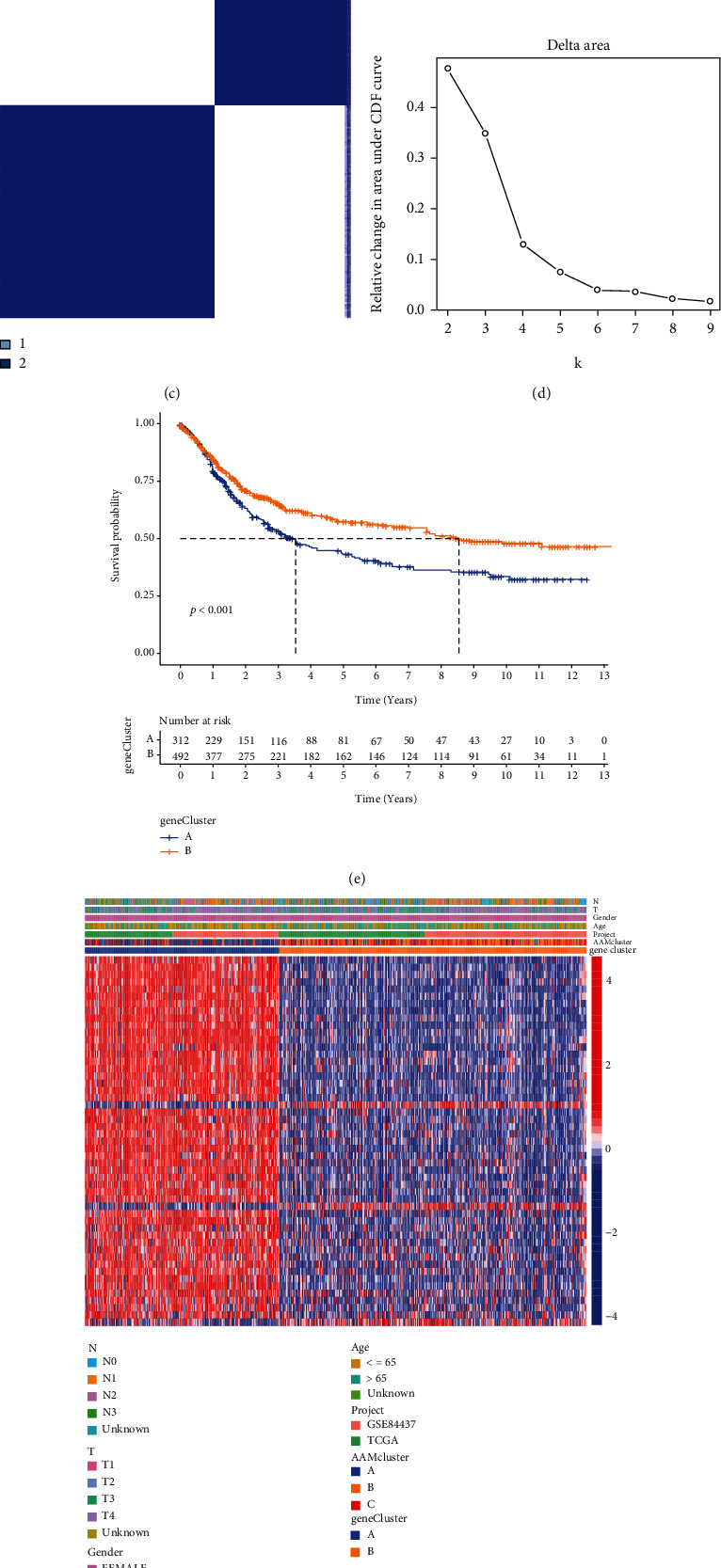
Generation of AAMs signatures. (a) Venn diagram showing overlapping genes of three distinct subgroups. (b) Results of GO enrichment. (c, d) Consensus matrix heatmap defining two gene clusters and their correlation area. (e) Kaplan–Meier curves showing the overall survival of gene clusters. (f) Heatmap of the clinical relevance of three AAM clusters and two geneClusters. (g) Gene expression levels of AAM-related genes in two geneClusters.

We discovered 65 overlapped genes in the three groups to further investigate the possible biological properties of AAM-related genes (Figure 4(a)). GO enrichment analysis revealed that these cluster-related genes were primarily enriched in biological processes associated with metastasis (Figure 4(b)). Afterward, we performed a uniCox analysis to determine the importance of these genes for survival. 51 genes were considered for the next analysis because they met *P* < 0.05 criteria (Table S4). Individuals were separated into 2 gene clusters (clusters A and B) based on prognostic genes to investigate this regulatory regime (Figures 4(c) and 4(d)). We identified that the OS time for cluster A patients was the shortest, while the OS time for cluster B patients was the best (Figure 4(e)).  Figure 4(f) shows a heatmap of the correlation between clusters and clinicopathological symptoms. The AAM gene clusters showed significant differences in the AAM gene expression, as predicted by the AAM subgroups (Figure 4(g)).

### 3.5. Prognostic AAM Score Construction and Validation

**Figure 5:**
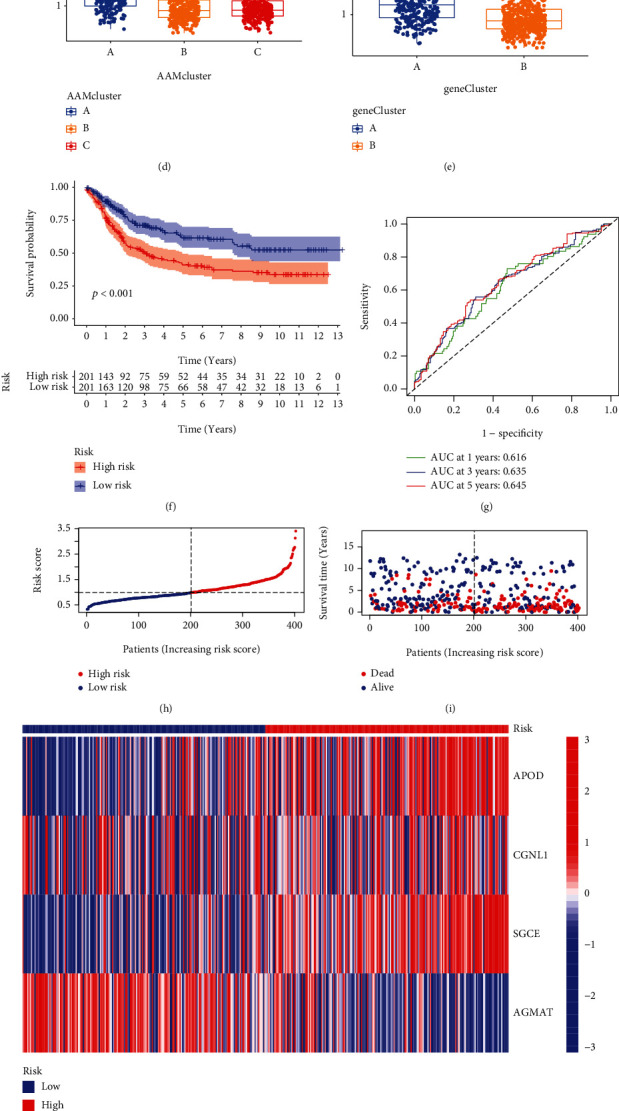
Four gene-based prognostic models were constructed using Lasso-Cox regression analysis. (a, b) The distribution of partial likelihood deviation of the Lasso coefficient preserves 4 variables when partial likelihood deviation reaches the minimum. (c) Alluvial diagram showing the connection between AAM clusters, gene clusters, and AAM score. (d) The level of the risk score in different AAM cluster subgroups. (e) The level of AAM score in different gene clusters. (f) The overall survival of AAM score. (g) ROC curves to predict the sensitivity and specificity of 1-, 3-, and 5-year survival according to the AAM score. (h, i) Ranked dot and scatter plots showing the AAM score distribution and patient survival status. (j) Heatmap showing four gene expression signatures in GC. (k, l) Establishment of nomogram and its performance verification.

The AAM score was derived from DEGs connected with clusters. The GC participants were randomly divided into two groups: a training group (*n* = 402) and a test group (*n* = 402) with a ratio of 1 : 1. LASSO Cox regression analysis built a prognostic gene model based on the 4 prognostic AAM genes (Figures 5(a) and 5(b)). This is the risk score: (0.099 × expression APOD) + (0.236 × expression CGNL1) + (0.213 × expression SGCE) + (0.164 × expression AGMAT).  Figure 5(c) shows the distribution of patients among the three subtypes of AAM, two gene subtypes, and two AAM score groups. Moreover, each gene cluster A (Figure 5(a)) and AAM cluster A (Figure 5(e)) had a high AAM score, whereas both gene cluster A (Figure 4(e)) and AAM cluster A (Figure 2(d)) had a poor prognosis. In the training sample, KM survival analysis revealed that OS rates were considerably lower in the high-score group than in the low-score group (log-rank test, *P* < 0.001) (Figure 5(f)). The ROC findings show AUC scores of 0.616, 0.635, and 0.645 for one, three, and five years, respectively, revealing that the signature's accuracy was adequate (Figure 5(g)). This finding also confirmed the results of our analysis. The hazard plot of the AAM value revealed that as the AAM score increased, OS time decreased, and death rates increased (Figures 5(h) and 5(i)).  Figure 5(j) also shows a heatmap of the chosen genes. Then, the nomogram plot showed that the AAM score might be a good tool for predicting long-term survival (Figure 5(k)). Calibration maps demonstrated that the nomogram technique was remarkably accurate, indicating that it can predict a patient's prognosis (Figure 5(l)).

### 3.6. AAM Score Association with TME, TMB, MSI, and CSC Score

**Figure 6:**
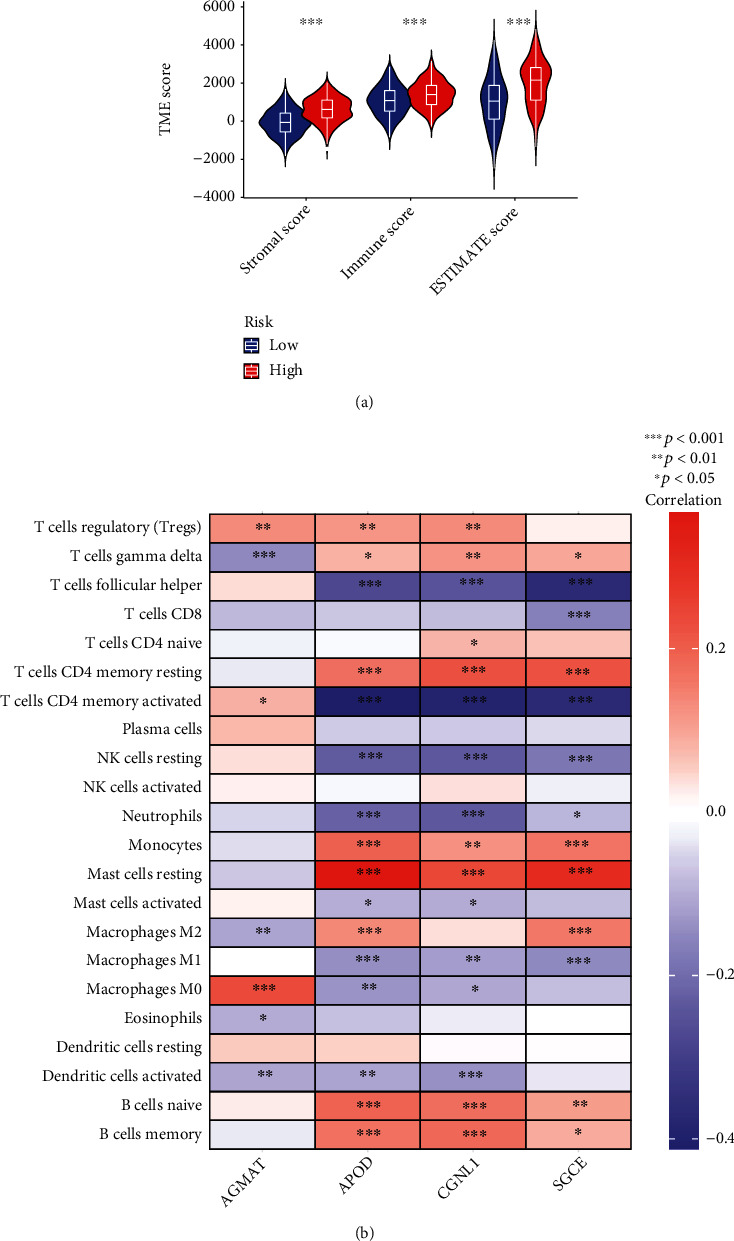
Evaluation of the TME between the two groups. (a) Correlations between AAM score and both immune and stromal scores. (b) Correlations between the abundance of immune cells and four genes in the proposed model.

We used the estimate package to determine the relationship between the AAM score and immune and stromal results. High AAM scores were closely linked to elevated immune scores, and high AAM scores were linked to increased stromal results (Figure 6(a)). The association between the four genes in the suggested model and the number of immune cells was also evaluated. Most immune cells were strongly linked to these genes (Figure 6(b)).

**Figure 7:**
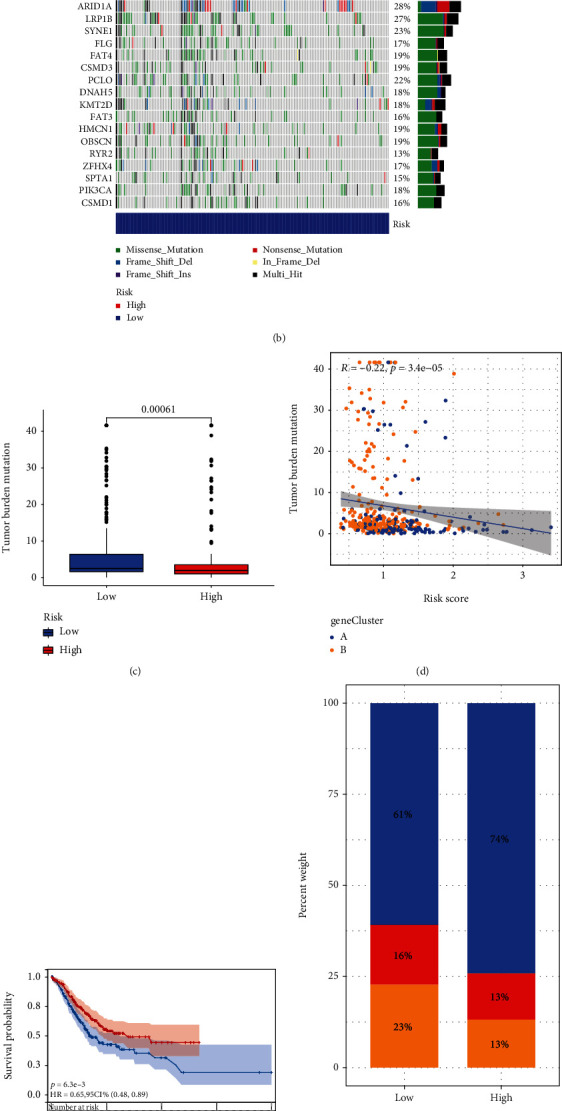
Comprehensive analysis of the AAM Score in GC. (a, b) The waterfall plot of somatic mutation features established with high and low AAM scores. (c) TMB in different AAM score groups. (d) Spearman's correlation analysis of the AAM score and TMB. (e) The Kaplan–Meier curves were used to perform survival analyses for patients with low and high TMB. (f, g) Relationships between AAM score and MSI. (h) Spearman's correlation analysis of the AAM score and RNAss. MSI: microsatellite instability; CSC: cancer stem cell; TMB: tumor mutation burden.

The “maftools” R software showed the distinctions in somatic mutation patterns between the higher and lower AAM score groups (Figures 7(a) and 7(b)). We discovered that TTN, TP53, and MUC16 mutation occurrences in GC patients in two risk categories were higher than or equivalent to 20%. Additionally, our results showed that the TMB was greater in the low-risk groups compared to the high-risk groups (Figure 7(c)), indicating that immunotherapy may be more beneficial for low-risk patients. Spearman's correlation analysis also demonstrated a negative relation between the AAM score and TMB (Figure 7(d)). We also performed a survival study across several TMB subgroups to examine the effect of TMB status on prognosis in GC patients. Individuals with elevated TMB had a better prognosis than those with reduced TMB (Figure 7(e)).

Moreover, correlation analysis revealed a significant relationship between a lower AAM score and the MSI-H condition, while a higher AAM score was associated with the microsatellite constant (MSS) condition (Figures 7(f) and 7(g)). We also integrated the AAM score and CSC index values to analyze any potential link between the AAM score and CSC in GC.  Figure 7(h) presents the findings of the linear correlation between the AAM score and the CSC index. We discovered that the AAM scoring was negatively related to the CSC index (R = 0.49, P < 2.2e − 16), indicating that GC cells with a lower AAM score exhibited stem cell features and a lower degree of cell differentiation.

### 3.7. Drug Sensitivity Testing

**Figure 8:**
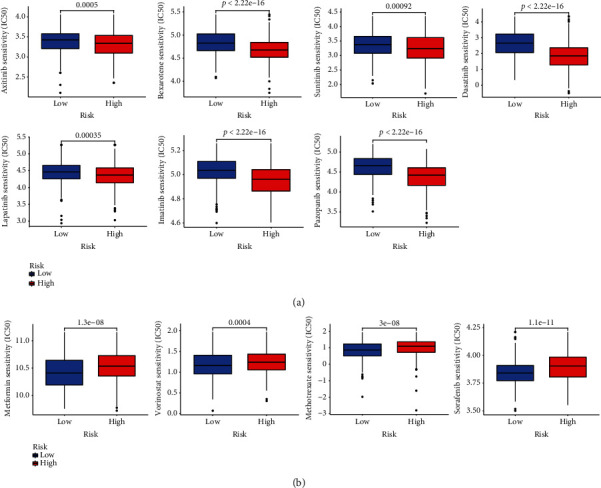
Relationships between AAM score and chemotherapeutic sensitivity. High IC50 of indicated chemotherapeutics drugs in low (a) and low (b) AAM score groups, respectively.

The IC_50_ of 98 drugs was measured in TCGA-STAD patients to determine the significance of the AAM value as an indicator of therapy response in GC patients. We found that individuals with high AAG scores may respond well to bexarotene and several targeted therapy agents, such as axitinib, sunitinib, dasatinib, lapatinib, imatinib, and pazopanib (Figures 8(a)). In contrast, individuals with low AAM scores may react better to metformin, vorinostat, methotrexate, and sorafenib (Figures 8(b)).

## 4. Discussion

GC is a highly heterogeneous malignant tumor that develops through the synergistic action of multiple mechanisms. Currently, the most common treatments for GC include surgery combined with immunotherapy, radiotherapy, chemotherapy, and targeted therapy [25]. However, survival outcomes for this cancer are far from satisfactory because of high recurrence and metastasis rates. Numerous studies have shown the essential function of amino acid metabolism in innate immunity and antitumor responses [26]. Additionally, most research has concentrated on a particular gene related to amino acid metabolism or a specific cell type in the TME. Therefore, the overall effect mediated by the combined action of multiple genes and the infiltration properties of the TME remains unknown. Moreover, our study can provide valuable information for the in-depth investigation. The outcomes of this research demonstrate alterations in transcription and AAM variations at the genetic level in GC.

Our study initially investigated the genetic alterations and AAM-related gene expressions using data from the TCGA-STAD and GSE84437 cohorts. While AAM gene mutation rates were lower, most prognosis-related genes were higher in GC patients. Then, we employed an unsupervised clustering approach to classify GC patients into three AAM subgroups. We found that clinical outcomes, immune infiltration, and function differed significantly among the three subgroups. Patients with subtype A exhibited a shorter OS and greater levels of immune cell infiltration than subtypes B and C. However, subtype A was considerably elevated in metastasis-related pathways. AAM cluster A was significantly enriched in innate immune cell infiltration, including activated B cells, activated CD4 T cells, activated CD8 T cells, activated DC, eosinophils, immature B cells, immature DC, MDSCs, macrophages, mast cells, NKT cells, NK cells, plasmacytoid DC cells, regulatory T cells, and T helper cells. Similar to the AAM clustering results, two genomic groupings with distinct clinical characteristics, immunological activities, and functions were discovered based on AAM-related genes. The AAM subgroups were quantified using LASSO Cox regression and the AAM score. Cluster A and gene cluster A, with the worst outcome measures, had the highest AAM value out of the three AAM clusters and two gene clusters. Amino acids can supply nitrogen and carbon for rapid tumor cell development and biosynthesis [27].

The AAM score was substantially related to the clinic pathological characteristics of GC. After adjusting for various factors, the results demonstrated that the AAM score was an independent predictor of survival outcomes in GC patients. ROC validated its predictive robustness over one, three, and five years of OS. Recently, risk scores associated with AAM have established clinical outcomes in GC patients. Moreover, the AAM score may provide useful prognostic information for patients. The aggregation of gene mutations leads to tumor development related to metabolic changes. According to our research, there are substantial variations between genetic modifications of individuals with low and high AAM scores. Elevated TMB was correlated with a better prognosis in GC patients, which is consistent with our results [28]. Clinical outcomes were substantially better in the low AAM score group compared to the low TMB group, indicating that the AAM score was a reliable predictor of immunotherapy performance. Immune checkpoint inhibitor treatment markedly enhanced outcomes in a comparatively higher percentage of MSI-H cancers than MSI-L/MSS tumors, providing significant and long-lasting reactions and survival advantages [29]. In this investigation, more MSI-H patients were found in the groups with a low AAM score and a better prognosis. AAM meets the cellular demands for maintaining redox homeostasis, energy generation, and biomass production and has been recognised as a key determinant of drug resistance in tumors [30, 31]. There is growing evidence that drug resistance in cancer cells can be overcome by suppressing or enhancing AAM and by depleting or supplementing amino acid availability [11, 32]. Currently, GC is slowly developing chemotherapeutics resistance. Finally, we explored the relationship between AAM score and chemotherapeutic drugs, which identified a novel insight for exploring tumor therapy treatment and avoiding the resistance of GC. The study identified drugs that may be effective for patients in various AAM score groups. Combining these drugs with the targeted AAM score may help reduce drug resistance and enhance clinical results. Our work uncovered the potential for repurposing “stale” chemotherapy drugs for new oncology indications.

We acknowledge that our research has several limitations. The first one was that all studies relied only on data obtained from public sources, and all samples were acquired retroactively in this research. The second limitation was that most datasets lacked data on crucial clinical characteristics like surgery, neoadjuvant chemotherapy, and chemoradiotherapy. The third was that more experimental studies should be conducted to confirm our findings.

## 5. Conclusion

In this study, we researched and identified several AAM genes that regulate TME, clinicopathological aspects, and prognosis. We also developed an AAM score to anticipate the prognosis and treatment sensitivity of GC patients, which assisted the development of more effective therapy regimens and paved the pathway for further research on the relationship between AAM-related genes and GC.

## Data Availability

The datasets analyzed for this study can be found in the TCGA-STAD project (http://www.cancer.gov/tcga) and GSE84337 from the GEO archive (https://www.ncbi.nlm.nih.gov/geo/).

## Supplementary Materials

Supplementary Materials Table S1: 101 amino acid metabolism-related denes. Table S2: clinical information of 871 gastric cancer patients. Table S3: univariate Cox regression analysis and the Kaplan–Meier analysis of 91 AAM genes associated with OS in GC patients. Table S4: univariate Cox regression analysis of 51 AAM genes associated with OS in GC patients.
