# Supplementary material for: Comprehensive Analysis to Reveal Amino Acid Metabolism-Associated Genes as a Prognostic Index in Gastric Cancer
Source: Mediators Inflamm. 2023 May 11;2023:3276319. doi: 10.1155/2023/3276319 (PMC10195167; doi:10.1155/2023/3276319)
Supplement: Supplementary Materials — Table S1: 101 amino acid metabolism-related denes. Table S2: clinical information of 871 gastric cancer patients. Table S3: univariate Cox regression analysis and the Kaplan–Meier analysis of 91 AAM genes associated with OS in GC patients. Table S4: univariate Cox regression analysis of 51 AAM genes associated with OS in GC patients. [file 3276319.f1.pdf]

Table S1: 101 Amino Acid Metabolism-related Genes
AMINO_ACID_AND_DERIVATIVE_METABOLIC_PROCESS
AARS1
ADI1
AIMP1
ALDH18A1
ALDH4A1
ALDH5A1
ALDH6A1
AMT
ARG1
ASL
ASMTL
ASPA
ASRGL1
ATF4
BAAT
BBOX1
BCAT1
BCKDHA
BCKDHB
BCKDK
BPHL
CDO1
COLQ
DARS1
DCT
DDAH1
DDAH2
DDO
DHPS
DIO1
DIO2
ETNK1
FAH
FARS2
FPGS
GAD1
GAD2
GAMT
GATM
GCLC
GCLM

GCSH
GGT1
GGT5
GLDC
GLS2
GLUD1
GLUD2
GOT1
GOT2
GSS
GSTZ1
HDC
HGD
HPD
HPRT1
IDO1
KARS1
KYAT1
MARS2
MAT1A
MAT2B
MCCC2
MSRA
MTHFR
NFS1
OAZ1
OAZ2
OTUB2
P4HB
PAH
PEPD
PLOD1
PRG3
PTS
PYCR1
QDPR
RARS1
SARS2
SCLY
SDS
SLC25A15
SLC3A1
SLC5A7
SLC6A14

SLC6A6
SLC7A2
SLC7A4
SLC7A5
SLC7A6
SLC7A7
SLC7A8
SLC7A9
SMS
SNCAIP
SULT1B1
TGFB2
TYR
WARS1
YARS1
YOD1

Table S2: Clinical information of 871 gastric cancer patients.

Id	Age	Gender	T	N	futime	fustat	Dataset
GSM2235556	61	MALE	T3	N1	2100	1	GSE84337
GSM2235557	47	FEMALE	T4	N3	690	1	GSE84337
GSM2235558	72	MALE	T4	N1	720	1	GSE84337
GSM2235559	78	MALE	T3	N2	1770	1	GSE84337
GSM2235560	58	MALE	T4	N0	360	1	GSE84337
GSM2235561	33	FEMALE	T4	N2	2550	0	GSE84337
GSM2235562	81	FEMALE	T4	N2	540	1	GSE84337
GSM2235563	60	FEMALE	T3	N2	420	1	GSE84337
GSM2235564	61	MALE	T3	N1	2520	0	GSE84337
GSM2235565	49	FEMALE	T4	N0	1950	1	GSE84337
GSM2235566	44	MALE	T4	N2	330	1	GSE84337
GSM2235567	51	MALE	T4	N2	1290	1	GSE84337
GSM2235568	58	FEMALE	T3	N1	2460	0	GSE84337
GSM2235569	79	MALE	T4	N1	2460	0	GSE84337
GSM2235570	77	MALE	T4	N0	2430	0	GSE84337
GSM2235571	85	FEMALE	T3	N1	2400	0	GSE84337
GSM2235572	68	FEMALE	T4	N1	2580	0	GSE84337
GSM2235573	52	MALE	T4	N2	2460	0	GSE84337
GSM2235574	72	MALE	T4	N2	330	1	GSE84337
GSM2235575	59	MALE	T3	N1	2580	0	GSE84337
GSM2235576	78	FEMALE	T3	N2	120	1	GSE84337
GSM2235577	32	FEMALE	T4	N0	570	1	GSE84337
GSM2235578	68	MALE	T4	N2	630	1	GSE84337
GSM2235579	74	MALE	T3	N1	510	1	GSE84337
GSM2235580	61	MALE	T4	N2	750	1	GSE84337

GSM2235582	65	MALE	T4	N0	390	1	GSE84337
GSM2235584	71	MALE	T4	N2	1710	1	GSE84337
GSM2235585	71	MALE	T3	N1	690	1	GSE84337
GSM2235586	71	MALE	T4	N1	2310	0	GSE84337
GSM2235587	72	MALE	T4	N1	1920	1	GSE84337
GSM2235588	56	MALE	T4	N1	1530	1	GSE84337
GSM2235589	66	MALE	T4	N2	2310	0	GSE84337
GSM2235593	56	MALE	T4	N0	2310	0	GSE84337
GSM2235595	74	MALE	T4	N1	450	1	GSE84337
GSM2235596	63	MALE	T3	N1	2250	0	GSE84337
GSM2235597	68	MALE	T2	N1	2520	0	GSE84337
GSM2235598	74	MALE	T3	N1	630	1	GSE84337
GSM2235599	62	MALE	T3	N2	2400	0	GSE84337
GSM2235600	53	MALE	T4	N2	2220	0	GSE84337
GSM2235601	79	MALE	T4	N2	2220	0	GSE84337
GSM2235602	61	FEMALE	T3	N1	2460	0	GSE84337
GSM2235603	60	FEMALE	T4	N1	2400	0	GSE84337
GSM2235604	75	MALE	T3	N1	2220	0	GSE84337
GSM2235605	54	FEMALE	T4	N1	2010	1	GSE84337
GSM2235606	38	MALE	T4	N2	2190	0	GSE84337
GSM2235607	65	MALE	T3	N2	180	1	GSE84337
GSM2235608	55	MALE	T3	N1	2400	0	GSE84337
GSM2235609	73	FEMALE	T4	N2	240	1	GSE84337
GSM2235610	76	MALE	T4	N2	690	1	GSE84337
GSM2235611	78	FEMALE	T4	N2	240	1	GSE84337
GSM2235612	69	MALE	T4	N2	870	1	GSE84337
GSM2235613	68	MALE	T2	N1	2490	0	GSE84337
GSM2235614	27	MALE	T4	N2	2310	0	GSE84337
GSM2235615	68	MALE	T3	N2	2310	0	GSE84337
GSM2235616	73	MALE	T4	N1	30	1	GSE84337
GSM2235617	54	MALE	T4	N2	2130	0	GSE84337
GSM2235618	73	FEMALE	T4	N2	2130	0	GSE84337
GSM2235619	55	FEMALE	T4	N1	2100	0	GSE84337
GSM2235620	65	MALE	T3	N2	2130	0	GSE84337
GSM2235621	63	MALE	T4	N2	990	1	GSE84337
GSM2235622	50	MALE	T3	N2	690	1	GSE84337
GSM2235623	56	MALE	T3	N1	630	1	GSE84337
GSM2235624	59	MALE	T3	N1	2310	0	GSE84337
GSM2235625	53	FEMALE	T4	N0	2100	0	GSE84337
GSM2235626	46	MALE	T4	N2	2310	0	GSE84337
GSM2235627	68	MALE	T3	N1	2100	0	GSE84337
GSM2235628	43	MALE	T3	N1	2220	0	GSE84337
GSM2235629	78	MALE	T4	N1	1080	1	GSE84337
GSM2235630	65	MALE	T4	N1	2100	0	GSE84337

GSM2235631	66	FEMALE	T4	N0	2070	0	GSE84337
GSM2235632	60	MALE	T3	N1	2280	0	GSE84337
GSM2235633	63	MALE	T4	N2	510	1	GSE84337
GSM2235634	64	FEMALE	T4	N1	2220	0	GSE84337
GSM2235635	73	MALE	T2	N2	2160	0	GSE84337
GSM2235636	32	FEMALE	T4	N2	2070	0	GSE84337
GSM2235637	75	FEMALE	T4	N0	2040	0	GSE84337
GSM2235695	42	FEMALE	T4	N1	3630	0	GSE84337
GSM2235696	60	MALE	T4	N1	3240	0	GSE84337
GSM2235697	49	MALE	T3	N0	1170	1	GSE84337
GSM2235698	34	MALE	T3	N3	690	1	GSE84337
GSM2235699	68	MALE	T4	N3	690	1	GSE84337
GSM2235700	59	MALE	T4	N2	3510	0	GSE84337
GSM2235701	61	MALE	T3	N0	3240	0	GSE84337
GSM2235702	74	FEMALE	T4	N1	540	1	GSE84337
GSM2235703	35	MALE	T4	N2	1290	1	GSE84337
GSM2235704	53	FEMALE	T4	N1	3270	0	GSE84337
GSM2235705	67	MALE	T4	N1	3420	0	GSE84337
GSM2235706	71	MALE	T4	N2	60	1	GSE84337
GSM2235707	63	FEMALE	T2	N1	3480	0	GSE84337
GSM2235708	66	MALE	T4	N1	3270	0	GSE84337
GSM2235709	54	FEMALE	T2	N0	3240	0	GSE84337
GSM2235710	54	MALE	T4	N0	390	1	GSE84337
GSM2235711	65	MALE	T4	N1	570	1	GSE84337
GSM2235712	70	FEMALE	T2	N0	3240	0	GSE84337
GSM2235713	59	MALE	T4	N0	3480	1	GSE84337
GSM2235714	61	MALE	T2	N1	1380	1	GSE84337
GSM2235715	58	MALE	T4	N1	3540	0	GSE84337
GSM2235716	46	MALE	T3	N1	3120	1	GSE84337
GSM2235717	43	FEMALE	T4	N3	90	1	GSE84337
GSM2235718	46	MALE	T4	N1	3570	0	GSE84337
GSM2235719	68	MALE	T4	N2	30	1	GSE84337
GSM2235720	56	MALE	T1	N0	3570	0	GSE84337
GSM2235721	41	MALE	T2	N1	3570	0	GSE84337
GSM2235722	63	FEMALE	T4	N2	1080	1	GSE84337
GSM2235723	52	MALE	T3	N2	3540	0	GSE84337
GSM2235724	74	MALE	T3	N3	300	1	GSE84337
GSM2235725	65	FEMALE	T4	N0	3660	0	GSE84337
GSM2235726	68	MALE	T4	N2	30	1	GSE84337
GSM2235727	71	MALE	T4	N3	3540	0	GSE84337
GSM2235728	40	FEMALE	T4	N1	1050	1	GSE84337
GSM2235729	67	MALE	T4	N2	150	1	GSE84337
GSM2235730	68	FEMALE	T1	N1	1470	1	GSE84337
GSM2235731	63	FEMALE	T2	N1	3540	0	GSE84337

GSM2235732	74	MALE	T2	N1	3540	0	GSE84337
GSM2235733	72	MALE	T3	N1	4320	0	GSE84337
GSM2235734	45	FEMALE	T4	N1	4320	0	GSE84337
GSM2235735	53	MALE	T3	N2	4350	0	GSE84337
GSM2235736	61	MALE	T4	N2	270	1	GSE84337
GSM2235737	51	MALE	T4	N2	1350	1	GSE84337
GSM2235738	59	FEMALE	T4	N2	4590	0	GSE84337
GSM2235739	66	MALE	T3	N2	420	1	GSE84337
GSM2235740	69	MALE	T3	N1	120	1	GSE84337
GSM2235741	49	MALE	T4	N2	3810	0	GSE84337
GSM2235742	62	MALE	T4	N2	90	1	GSE84337
GSM2235743	64	FEMALE	T4	N1	120	1	GSE84337
GSM2235744	38	FEMALE	T4	N1	1470	1	GSE84337
GSM2235745	53	MALE	T4	N2	3180	0	GSE84337
GSM2235746	53	MALE	T3	N1	3330	0	GSE84337
GSM2235747	53	FEMALE	T4	N2	360	1	GSE84337
GSM2235748	80	MALE	T4	N2	60	1	GSE84337
GSM2235749	65	MALE	T4	N3	1170	1	GSE84337
GSM2235750	70	MALE	T4	N1	210	1	GSE84337
GSM2235751	40	MALE	T3	N1	3030	0	GSE84337
GSM2235752	42	MALE	T4	N1	4470	0	GSE84337
GSM2235753	72	MALE	T4	N1	930	1	GSE84337
GSM2235754	37	FEMALE	T4	N1	4140	0	GSE84337
GSM2235755	62	FEMALE	T4	N1	2610	1	GSE84337
GSM2235756	48	FEMALE	T4	N2	4200	0	GSE84337
GSM2235757	44	MALE	T4	N2	1200	1	GSE84337
GSM2235758	42	MALE	T4	N2	390	1	GSE84337
GSM2235759	54	MALE	T4	N2	360	1	GSE84337
GSM2235760	64	MALE	T4	N2	600	1	GSE84337
GSM2235761	63	FEMALE	T4	N1	4020	0	GSE84337
GSM2235762	65	MALE	T4	N2	1170	1	GSE84337
GSM2235763	74	FEMALE	T4	N1	3270	1	GSE84337
GSM2235764	58	MALE	T4	N2	270	1	GSE84337
GSM2235765	68	MALE	T4	N1	4140	0	GSE84337
GSM2235766	61	MALE	T4	N2	450	1	GSE84337
GSM2235767	53	MALE	T4	N2	540	1	GSE84337
GSM2235768	46	MALE	T4	N1	4320	0	GSE84337
GSM2235769	64	MALE	T3	N2	660	1	GSE84337
GSM2235770	85	MALE	T3	N1	210	1	GSE84337
GSM2235771	53	MALE	T4	N1	4290	0	GSE84337
GSM2235772	60	MALE	T4	N2	1800	1	GSE84337
GSM2235773	30	MALE	T4	N2	510	1	GSE84337
GSM2235774	47	MALE	T4	N1	360	1	GSE84337
GSM2235775	60	MALE	T4	N1	3600	0	GSE84337

GSM2235776	80	MALE	T4	N0	600	1	GSE84337
GSM2235777	49	FEMALE	T4	N1	3450	1	GSE84337
GSM2235778	61	MALE	T4	N3	690	1	GSE84337
GSM2235779	63	MALE	T4	N0	1470	1	GSE84337
GSM2235780	75	MALE	T4	N3	0	1	GSE84337
GSM2235781	56	MALE	T1	N0	3450	0	GSE84337
GSM2235782	62	MALE	T4	N2	120	1	GSE84337
GSM2235783	71	MALE	T3	N1	2250	1	GSE84337
GSM2235784	80	MALE	T2	N0	3090	1	GSE84337
GSM2235785	64	FEMALE	T3	N0	3420	0	GSE84337
GSM2235786	50	FEMALE	T4	N1	3240	0	GSE84337
GSM2235787	58	FEMALE	T4	N2	750	1	GSE84337
GSM2235788	56	MALE	T2	N0	3510	0	GSE84337
GSM2235789	64	MALE	T4	N2	1110	1	GSE84337
GSM2235790	67	FEMALE	T4	N0	3570	0	GSE84337
GSM2235791	36	FEMALE	T4	N1	750	1	GSE84337
GSM2235792	60	FEMALE	T4	N1	2760	1	GSE84337
GSM2235793	83	MALE	T3	N2	2610	1	GSE84337
GSM2235794	55	MALE	T1	N1	3630	0	GSE84337
GSM2235795	73	FEMALE	T4	N0	3630	0	GSE84337
GSM2235796	62	MALE	T4	N3	1320	1	GSE84337
GSM2235797	79	FEMALE	T3	N0	3450	0	GSE84337
GSM2235798	76	MALE	T4	N2	750	1	GSE84337
GSM2235799	62	MALE	T4	N3	360	1	GSE84337
GSM2235800	62	MALE	T3	N1	3420	0	GSE84337
GSM2235801	50	MALE	T2	N1	3390	0	GSE84337
GSM2235802	60	MALE	T1	N0	3180	0	GSE84337
GSM2235803	46	MALE	T3	N0	3210	0	GSE84337
GSM2235804	64	MALE	T4	N0	3420	0	GSE84337
GSM2235805	61	FEMALE	T4	N1	150	1	GSE84337
GSM2235806	55	MALE	T4	N0	3030	1	GSE84337
GSM2235807	70	MALE	T4	N3	990	1	GSE84337
GSM2235808	59	MALE	T4	N1	690	1	GSE84337
GSM2235809	58	MALE	T4	N1	4380	0	GSE84337
GSM2235810	54	MALE	T4	N1	210	1	GSE84337
GSM2235811	55	MALE	T4	N2	360	1	GSE84337
GSM2235812	68	MALE	T4	N3	1020	1	GSE84337
GSM2235813	64	MALE	T3	N0	780	1	GSE84337
GSM2235814	66	MALE	T4	N3	3390	0	GSE84337
GSM2235815	71	FEMALE	T4	N2	3390	0	GSE84337
GSM2235816	48	MALE	T4	N3	120	1	GSE84337
GSM2235817	68	FEMALE	T4	N1	1650	1	GSE84337
GSM2235818	74	FEMALE	T4	N3	300	1	GSE84337
GSM2235819	68	MALE	T4	N1	630	1	GSE84337

GSM2235820	75	MALE	T4	N1	720	1	GSE84337
GSM2235821	67	MALE	T4	N1	3360	0	GSE84337
GSM2235822	55	MALE	T3	N1	3360	0	GSE84337
GSM2235823	32	FEMALE	T4	N1	270	1	GSE84337
GSM2235824	49	FEMALE	T4	N2	2370	1	GSE84337
GSM2235825	69	MALE	T4	N0	690	1	GSE84337
GSM2235826	79	MALE	T3	N2	330	1	GSE84337
GSM2235827	47	MALE	T4	N2	1290	1	GSE84337
GSM2235828	55	MALE	T4	N1	3210	0	GSE84337
GSM2235829	52	MALE	T4	N0	3270	0	GSE84337
GSM2235830	75	MALE	T4	N2	2940	0	GSE84337
GSM2235831	81	MALE	T4	N3	1170	1	GSE84337
GSM2235837	56	FEMALE	T2	N0	3390	0	GSE84337
GSM2235838	65	MALE	T4	N3	120	1	GSE84337
GSM2235839	63	MALE	T3	N1	3030	1	GSE84337
GSM2235840	71	MALE	T2	N0	2760	1	GSE84337
GSM2235841	84	FEMALE	T4	N1	480	1	GSE84337
GSM2235842	72	MALE	T4	N3	420	1	GSE84337
GSM2235843	60	MALE	T2	N1	390	1	GSE84337
GSM2235844	54	FEMALE	T4	N2	3060	0	GSE84337
GSM2235845	51	MALE	T4	N2	930	1	GSE84337
GSM2235846	67	MALE	T4	N2	2760	1	GSE84337
GSM2235847	53	FEMALE	T4	N1	3330	0	GSE84337
GSM2235848	52	MALE	T4	N2	1200	1	GSE84337
GSM2235849	61	FEMALE	T4	N2	30	1	GSE84337
GSM2235850	51	MALE	T2	N1	3480	0	GSE84337
GSM2235851	81	MALE	T1	N0	2790	1	GSE84337
GSM2235852	69	MALE	T4	N3	1440	1	GSE84337
GSM2235853	58	FEMALE	T2	N1	3330	0	GSE84337
GSM2235854	66	FEMALE	T4	N3	3330	0	GSE84337
GSM2235855	68	MALE	T2	N1	3300	0	GSE84337
GSM2235862	66	MALE	T4	N0	2550	1	GSE84337
GSM2235863	67	MALE	T4	N0	3990	0	GSE84337
GSM2235864	54	MALE	T4	N1	360	1	GSE84337
GSM2235865	44	MALE	T4	N1	990	1	GSE84337
GSM2235866	79	FEMALE	T4	N2	3870	0	GSE84337
GSM2235867	69	FEMALE	T4	N2	3750	0	GSE84337
GSM2235878	51	FEMALE	T4	N3	3750	0	GSE84337
GSM2235879	68	FEMALE	T3	N1	180	1	GSE84337
GSM2235880	67	FEMALE	T4	N2	510	1	GSE84337
GSM2235881	72	MALE	T4	N0	3750	0	GSE84337
GSM2235882	55	MALE	T4	N1	390	1	GSE84337
GSM2235883	66	FEMALE	T4	N2	570	1	GSE84337
GSM2235884	44	MALE	T4	N0	3780	0	GSE84337

GSM2235885	73	FEMALE	T4	N1	3720	0	GSE84337
GSM2235886	76	FEMALE	T2	N1	1530	1	GSE84337
GSM2235887	81	MALE	T3	N2	2370	1	GSE84337
GSM2235888	36	FEMALE	T4	N1	2040	1	GSE84337
GSM2235889	66	MALE	T4	N1	2790	1	GSE84337
GSM2235899	48	MALE	T4	N1	1020	1	GSE84337
GSM2235900	46	MALE	T4	N1	3810	0	GSE84337
GSM2235901	66	FEMALE	T3	N1	1740	1	GSE84337
GSM2235902	34	MALE	T4	N1	1020	1	GSE84337
GSM2235903	50	MALE	T4	N1	3930	0	GSE84337
GSM2235904	62	MALE	T4	N1	630	1	GSE84337
GSM2235905	63	MALE	T4	N1	60	1	GSE84337
GSM2235906	70	FEMALE	T4	N1	3600	1	GSE84337
GSM2235907	77	FEMALE	T4	N0	3690	0	GSE84337
GSM2235908	32	FEMALE	T3	N0	3690	0	GSE84337
GSM2235909	63	MALE	T4	N2	540	1	GSE84337
GSM2235910	56	MALE	T4	N1	4170	0	GSE84337
GSM2235921	57	MALE	T2	N2	4170	0	GSE84337
GSM2235922	68	MALE	T4	N1	4140	0	GSE84337
GSM2235923	77	FEMALE	T4	N1	420	1	GSE84337
GSM2235924	54	MALE	T4	N2	4050	0	GSE84337
GSM2235925	64	MALE	T4	N1	4020	0	GSE84337
GSM2235926	47	MALE	T4	N2	4200	0	GSE84337
GSM2235927	65	MALE	T4	N1	0	1	GSE84337
GSM2235928	56	FEMALE	T4	N1	3960	0	GSE84337
GSM2235929	74	MALE	T4	N2	1800	1	GSE84337
GSM2235930	63	MALE	T4	N2	2220	1	GSE84337
GSM2235931	34	MALE	T4	N1	3960	0	GSE84337
GSM2235932	71	FEMALE	T4	N2	1770	0	GSE84337
GSM2235933	57	FEMALE	T4	N0	1230	0	GSE84337
GSM2235934	63	MALE	T4	N2	1410	0	GSE84337
GSM2235935	65	MALE	T4	N2	570	1	GSE84337
GSM2235936	63	FEMALE	T1	N0	1260	0	GSE84337
GSM2235937	54	FEMALE	T4	N2	1110	0	GSE84337
GSM2235938	62	FEMALE	T1	N0	2760	0	GSE84337
GSM2235939	42	FEMALE	T2	N0	1350	0	GSE84337
GSM2235940	63	MALE	T4	N2	990	1	GSE84337
GSM2235941	36	FEMALE	T2	N1	1230	0	GSE84337
GSM2235942	67	MALE	T1	N0	1260	0	GSE84337
GSM2235943	65	MALE	T4	N1	3810	0	GSE84337
GSM2235944	47	MALE	T3	N0	3780	0	GSE84337
GSM2235945	59	FEMALE	T4	N1	1110	1	GSE84337
GSM2235946	55	MALE	T4	N2	3930	0	GSE84337
GSM2235947	62	MALE	T4	N1	3870	0	GSE84337

GSM2235948	36	MALE	T4	N1	3960	0	GSE84337
GSM2235949	43	MALE	T4	N1	3960	0	GSE84337
GSM2235950	45	MALE	T4	N1	3840	0	GSE84337
GSM2235951	61	FEMALE	T4	N1	150	1	GSE84337
GSM2235952	56	FEMALE	T4	N1	30	1	GSE84337
GSM2235953	58	FEMALE	T4	N1	300	1	GSE84337
GSM2235954	39	FEMALE	T3	N1	4470	0	GSE84337
GSM2235955	48	MALE	T4	N2	4470	0	GSE84337
GSM2235956	68	FEMALE	T4	N2	4050	1	GSE84337
GSM2235957	69	FEMALE	T3	N2	4440	0	GSE84337
GSM2235958	55	MALE	T4	N2	4560	0	GSE84337
GSM2235959	61	MALE	T3	N2	4320	0	GSE84337
GSM2235960	66	MALE	T4	N1	4260	0	GSE84337
GSM2235961	55	MALE	T4	N1	4230	0	GSE84337
GSM2235962	64	MALE	T4	N1	810	1	GSE84337
GSM2235963	58	FEMALE	T4	N2	810	1	GSE84337
GSM2235964	64	MALE	T4	N2	660	1	GSE84337
GSM2235965	58	MALE	T3	N1	1890	1	GSE84337
GSM2235966	40	MALE	T4	N3	990	0	GSE84337
GSM2235967	61	MALE	T4	N2	300	1	GSE84337
GSM2235968	52	MALE	T2	N0	1770	0	GSE84337
GSM2235969	72	FEMALE	T2	N1	1920	0	GSE84337
GSM2235970	66	FEMALE	T4	N2	540	1	GSE84337
GSM2235971	45	MALE	T3	N2	1890	0	GSE84337
GSM2235972	67	MALE	T4	N3	1710	0	GSE84337
GSM2235973	59	FEMALE	T3	N0	1860	0	GSE84337
GSM2235974	63	MALE	T4	N2	360	1	GSE84337
GSM2235975	59	MALE	T3	N3	1830	0	GSE84337
GSM2235976	67	FEMALE	T3	N1	1650	0	GSE84337
GSM2235977	72	MALE	T4	N2	210	1	GSE84337
GSM2235978	65	MALE	T4	N2	1020	0	GSE84337
GSM2235979	54	FEMALE	T2	N1	1260	0	GSE84337
GSM2235980	66	MALE	T4	N1	180	1	GSE84337
GSM2235981	69	MALE	T4	N1	1080	0	GSE84337
GSM2235982	51	MALE	T3	N2	1110	0	GSE84337
GSM2235983	46	MALE	T3	N1	1140	0	GSE84337
GSM2235984	69	MALE	T4	N3	240	1	GSE84337
GSM2235985	69	FEMALE	T4	N3	1050	0	GSE84337
GSM2235986	53	FEMALE	T3	N3	1050	0	GSE84337
GSM2235987	69	MALE	T4	N1	1050	0	GSE84337
GSM2235988	54	FEMALE	T1	N0	2100	0	GSE84337
GSM2235991	77	MALE	T3	N2	960	1	GSE84337
GSM2235992	59	MALE	T4	N1	3840	0	GSE84337
GSM2235993	27	MALE	T3	N1	3780	0	GSE84337

GSM2235994	42	FEMALE	T3	N1	3690	0	GSE84337
GSM2235995	57	FEMALE	T3	N2	300	1	GSE84337
GSM2235996	53	FEMALE	T4	N1	180	1	GSE84337
GSM2235997	74	MALE	T4	N1	3840	0	GSE84337
GSM2235998	68	MALE	T4	N1	3660	0	GSE84337
GSM2235999	60	MALE	T4	N1	2850	1	GSE84337
GSM2236000	64	FEMALE	T3	N1	3660	0	GSE84337
GSM2236001	48	FEMALE	T4	N2	3720	0	GSE84337
GSM2236002	68	FEMALE	T4	N1	3870	0	GSE84337
GSM2236003	55	MALE	T4	N1	3780	0	GSE84337
GSM2236004	66	MALE	T4	N1	1380	1	GSE84337
GSM2236005	72	FEMALE	T4	N2	270	1	GSE84337
GSM2236006	42	MALE	T4	N1	720	1	GSE84337
GSM2236007	76	FEMALE	T4	N1	630	1	GSE84337
GSM2236008	64	FEMALE	T3	N1	900	1	GSE84337
GSM2236009	66	MALE	T4	N1	810	1	GSE84337
GSM2236010	61	MALE	T4	N1	1140	1	GSE84337
GSM2236011	64	MALE	T3	N2	780	1	GSE84337
GSM2236012	33	MALE	T4	N3	90	1	GSE84337
GSM2236013	67	FEMALE	T2	N1	1590	1	GSE84337
GSM2236014	68	MALE	T3	N0	3150	1	GSE84337
GSM2236015	66	MALE	T4	N2	3300	0	GSE84337
GSM2236016	53	MALE	T4	N2	600	1	GSE84337
GSM2236017	61	MALE	T4	N2	510	1	GSE84337
GSM2236018	36	MALE	T4	N2	3480	0	GSE84337
GSM2236019	77	MALE	T3	N2	2760	1	GSE84337
GSM2236020	70	FEMALE	T4	N0	3270	0	GSE84337
GSM2236021	32	MALE	T4	N3	1530	1	GSE84337
GSM2236022	53	MALE	T4	N3	3120	0	GSE84337
GSM2236023	61	FEMALE	T2	N0	3240	0	GSE84337
GSM2236024	54	MALE	T3	N1	3090	0	GSE84337
GSM2236025	68	FEMALE	T3	N1	1170	1	GSE84337
GSM2236026	65	MALE	T3	N1	1920	1	GSE84337
GSM2236027	80	FEMALE	T4	N1	3720	0	GSE84337
GSM2236028	68	MALE	T3	N0	2850	1	GSE84337
GSM2236029	44	MALE	T2	N1	4140	0	GSE84337
GSM2236030	63	MALE	T4	N0	4140	0	GSE84337
GSM2236031	52	FEMALE	T4	N0	4020	0	GSE84337
GSM2236032	44	MALE	T4	N0	4020	0	GSE84337
GSM2236033	69	MALE	T3	N0	3600	0	GSE84337
GSM2236034	55	MALE	T4	N0	3660	0	GSE84337
GSM2236036	62	MALE	T1	N1	1230	0	GSE84337
GSM2236037	64	FEMALE	T4	N1	1230	0	GSE84337
GSM2236038	65	MALE	T4	N2	1200	0	GSE84337

GSM2236039	56	MALE	T2	N0	1260	0	GSE84337
GSM2236040	71	FEMALE	T3	N1	1110	0	GSE84337
GSM2236041	50	MALE	T4	N1	1230	0	GSE84337
GSM2236042	69	FEMALE	T4	N2	1110	0	GSE84337
GSM2236043	48	MALE	T3	N1	1200	0	GSE84337
GSM2236044	48	MALE	T2	N2	4230	0	GSE84337
GSM2236045	63	MALE	T4	N1	4830	0	GSE84337
GSM2236046	64	MALE	T4	N1	1050	1	GSE84337
GSM2236047	49	FEMALE	T4	N2	1470	1	GSE84337
GSM2236048	64	MALE	T3	N0	3060	0	GSE84337
GSM2236049	44	FEMALE	T4	N1	990	1	GSE84337
GSM2236050	65	MALE	T3	N1	3030	0	GSE84337
GSM2236051	34	MALE	T4	N2	1140	1	GSE84337
GSM2236052	61	MALE	T4	N1	3480	0	GSE84337
GSM2236053	41	FEMALE	T4	N2	660	1	GSE84337
GSM2236054	49	FEMALE	T4	N0	2370	1	GSE84337
GSM2236055	55	MALE	T4	N0	3990	0	GSE84337
GSM2236056	54	MALE	T4	N0	3840	0	GSE84337
GSM2236057	62	MALE	T2	N1	1110	1	GSE84337
GSM2236058	62	MALE	T2	N1	4080	0	GSE84337
GSM2236059	75	FEMALE	T2	N1	4110	0	GSE84337
GSM2236060	47	MALE	T4	N3	1110	1	GSE84337
GSM2236061	68	FEMALE	T2	N1	3870	0	GSE84337
GSM2236062	69	MALE	T3	N1	3870	0	GSE84337
GSM2236063	48	FEMALE	T2	N1	3930	0	GSE84337
GSM2236064	70	MALE	T4	N0	2310	1	GSE84337
GSM2236065	58	MALE	T4	N2	3570	0	GSE84337
GSM2236066	63	FEMALE	T4	N1	3810	0	GSE84337
GSM2236067	47	MALE	T4	N2	510	1	GSE84337
GSM2236068	44	MALE	T4	N0	1260	1	GSE84337
GSM2236069	69	MALE	T3	N1	3450	0	GSE84337
GSM2236070	71	MALE	T3	N1	3720	0	GSE84337
GSM2236071	54	FEMALE	T4	N1	3420	0	GSE84337
GSM2236072	49	MALE	T3	N3	990	1	GSE84337
GSM2236073	60	MALE	T4	N2	660	1	GSE84337
GSM2236074	62	MALE	T2	N1	4290	0	GSE84337
GSM2236075	44	MALE	T4	N1	1170	1	GSE84337
GSM2236076	63	MALE	T3	N1	1050	0	GSE84337
GSM2236077	68	MALE	T4	N2	1140	0	GSE84337
GSM2236078	60	MALE	T4	N0	4260	0	GSE84337
GSM2236079	67	MALE	T2	N1	4230	0	GSE84337
GSM2236080	62	MALE	T4	N0	420	1	GSE84337
GSM2236082	53	FEMALE	T4	N0	3930	0	GSE84337
GSM2236083	64	FEMALE	T4	N2	210	1	GSE84337

GSM2236084	59	FEMALE	T3	N2	3840	0	GSE84337
GSM2236085	55	MALE	T4	N0	4560	0	GSE84337
GSM2236086	36	FEMALE	T4	N0	4470	0	GSE84337
GSM2236087	68	MALE	T3	N1	4650	0	GSE84337
GSM2236088	61	FEMALE	T4	N0	1020	1	GSE84337
GSM2236089	69	FEMALE	T4	N0	4320	0	GSE84337
GSM2236090	86	MALE	T4	N0	930	1	GSE84337
GSM2236091	77	MALE	T3	N0	3690	1	GSE84337
GSM2236092	58	MALE	T4	N0	4470	0	GSE84337
GSM2236093	59	MALE	T3	N0	4530	0	GSE84337
GSM2236094	64	MALE	T4	N0	1110	1	GSE84337
GSM2236095	51	MALE	T4	N0	4500	0	GSE84337
TCGA-3M-AB46	70	MALE	T2	N0	1765	0	TCGA
TCGA-B7-5816	51	FEMALE	T4	N0	812	0	TCGA
TCGA-B7-5818	62	MALE	T2	N0	356	0	TCGA
TCGA-B7-A5TI	52	MALE	T4	N3	595	0	TCGA
TCGA-B7-A5TJ	74	MALE	T4	unknow	335	0	TCGA
TCGA-B7-A5TK	51	MALE	T4	unknow	288	0	TCGA
TCGA-B7-A5TN	60	MALE	T4	unknow	287	0	TCGA
TCGA-BR-4183	55	FEMALE	T3	N1	201	1	TCGA
TCGA-BR-4184	70	MALE	T3	N1	212	1	TCGA
TCGA-BR-4187	56	MALE	unknow	unknow	141	1	TCGA
TCGA-BR-4188	53	FEMALE	unknow	N1	226	1	TCGA
TCGA-BR-4191	72	MALE	unknow	N1	558	1	TCGA
TCGA-BR-4201	66	FEMALE	T2	unknow	940	1	TCGA
TCGA-BR-4253	80	FEMALE	T3	N1	124	1	TCGA
TCGA-BR-4255	76	FEMALE	T3	N1	124	1	TCGA
TCGA-BR-4256	80	MALE	unknow	N1	284	1	TCGA
TCGA-BR-4257	79	FEMALE	unknow	unknow	294	1	TCGA
TCGA-BR-4267	51	MALE	T2	N0	188	1	TCGA
TCGA-BR-4279	43	MALE	T2	N1	291	1	TCGA
TCGA-BR-4280	78	FEMALE	T2	N1	201	1	TCGA
TCGA-BR-4292	73	FEMALE	T1	unknow	0	0	TCGA
TCGA-BR-4294	65	MALE	T2	unknow	0	0	TCGA
TCGA-BR-4357	58	MALE	T3	unknow	0	0	TCGA
TCGA-BR-4361	66	FEMALE	T4	N0	0	0	TCGA
TCGA-BR-4362	74	FEMALE	unknow	N1	0	0	TCGA
TCGA-BR-4363	60	FEMALE	T3	unknow	0	0	TCGA
TCGA-BR-4366	87	MALE	unknow	unknow	0	0	TCGA
TCGA-BR-4367	78	MALE	unknow	N1	0	0	TCGA
TCGA-BR-4368	78	FEMALE	T4	N2	0	0	TCGA
TCGA-BR-4369	75	MALE	unknow	unknow	0	0	TCGA
TCGA-BR-4370	74	FEMALE	unknow	unknow	0	0	TCGA
TCGA-BR-4371	71	FEMALE	T2	unknow	0	0	TCGA

TCGA-BR-6452	78	FEMALE	T3	N0	1055	0	TCGA
TCGA-BR-6453	54	MALE	T2	N1	485	0	TCGA
TCGA-BR-6454	58	MALE	T3	N0	0	0	TCGA
TCGA-BR-6455	59	MALE	T3	N1	422	1	TCGA
TCGA-BR-6456	74	FEMALE	T3	N1	526	1	TCGA
TCGA-BR-6457	69	MALE	T3	N0	416	0	TCGA
TCGA-BR-6458	57	FEMALE	T3	N1	588	1	TCGA
TCGA-BR-6563	60	MALE	T3	N1	1190	0	TCGA
TCGA-BR-6564	46	FEMALE	T3	N2	794	1	TCGA
TCGA-BR-6565	67	MALE	T4	N0	279	1	TCGA
TCGA-BR-6566	64	FEMALE	T3	N0	997	0	TCGA
TCGA-BR-6705	68	FEMALE	T3	N3	779	1	TCGA
TCGA-BR-6706	63	MALE	T3	N1	549	1	TCGA
TCGA-BR-6707	75	MALE	T3	N0	605	1	TCGA
TCGA-BR-6709	57	FEMALE	T3	N3	370	1	TCGA
TCGA-BR-6710	41	MALE	T2	N0	273	0	TCGA
TCGA-BR-6801	70	MALE	T3	N0	1223	0	TCGA
TCGA-BR-6802	65	MALE	T3	N2	940	0	TCGA
TCGA-BR-6803	54	FEMALE	T3	N0	949	0	TCGA
TCGA-BR-6852	64	FEMALE	T3	N0	1367	0	TCGA
TCGA-BR-7196	64	MALE	T3	N3	666	0	TCGA
TCGA-BR-7197	69	MALE	T3	N0	280	0	TCGA
TCGA-BR-7703	81	MALE	T1	N0	1100	0	TCGA
TCGA-BR-7704	69	FEMALE	T3	N0	1072	0	TCGA
TCGA-BR-7707	69	FEMALE	T2	N0	1090	0	TCGA
TCGA-BR-7715	65	MALE	T3	N0	1023	0	TCGA
TCGA-BR-7716	62	FEMALE	T3	N1	1210	0	TCGA
TCGA-BR-7717	63	MALE	T4	N1	552	1	TCGA
TCGA-BR-7722	62	MALE	T3	N1	466	1	TCGA
TCGA-BR-7723	59	MALE	T3	N3	874	1	TCGA
TCGA-BR-7851	74	MALE	T4	N0	378	0	TCGA
TCGA-BR-7901	74	MALE	T3	N1	105	1	TCGA
TCGA-BR-7957	50	FEMALE	T3	N3	276	1	TCGA
TCGA-BR-7958	60	MALE	T4	N0	899	0	TCGA
TCGA-BR-7959	59	MALE	T4	N1	1010	0	TCGA
TCGA-BR-8058	53	FEMALE	T4	N2	1133	0	TCGA
TCGA-BR-8059	72	MALE	T4	N1	439	1	TCGA
TCGA-BR-8060	75	FEMALE	T2	N2	348	1	TCGA
TCGA-BR-8077	58	FEMALE	T4	N1	21	0	TCGA
TCGA-BR-8078	70	FEMALE	T4	N0	900	0	TCGA
TCGA-BR-8080	72	FEMALE	T4	N3	292	1	TCGA
TCGA-BR-8081	71	FEMALE	T4	N0	981	0	TCGA
TCGA-BR-8284	72	FEMALE	T4	N3	245	1	TCGA
TCGA-BR-8285	57	FEMALE	T4	N3	17	1	TCGA

TCGA-BR-8286	49	MALE	T3	N0	895	0	TCGA
TCGA-BR-8289	57	MALE	T4	N3	81	1	TCGA
TCGA-BR-8291	61	MALE	T3	N1	607	1	TCGA
TCGA-BR-8295	60	FEMALE	T4	N0	67	1	TCGA
TCGA-BR-8296	58	FEMALE	T4	N2	474	1	TCGA
TCGA-BR-8297	58	MALE	T4	N3	225	0	TCGA
TCGA-BR-8360	66	MALE	T3	N0	188	0	TCGA
TCGA-BR-8361	71	FEMALE	T4	N2	946	0	TCGA
TCGA-BR-8362	65	MALE	T4	N3	398	1	TCGA
TCGA-BR-8363	73	FEMALE	T2	N0	8	1	TCGA
TCGA-BR-8364	42	FEMALE	T4	N2	675	0	TCGA
TCGA-BR-8365	70	FEMALE	T3	N0	533	1	TCGA
TCGA-BR-8366	80	FEMALE	T3	N0	29	0	TCGA
TCGA-BR-8367	55	MALE	T3	N3	801	1	TCGA
TCGA-BR-8368	84	FEMALE	T2	N0	131	0	TCGA
TCGA-BR-8369	76	FEMALE	T3	N3	427	0	TCGA
TCGA-BR-8370	64	MALE	T3	N3	101	0	TCGA
TCGA-BR-8371	62	MALE	T3	N3	359	1	TCGA
TCGA-BR-8372	63	MALE	T4	N3	951	0	TCGA
TCGA-BR-8373	65	FEMALE	T4	N1	450	0	TCGA
TCGA-BR-8380	55	MALE	T4	N3	21	0	TCGA
TCGA-BR-8381	51	MALE	T3	N1	224	0	TCGA
TCGA-BR-8382	67	FEMALE	T4	N3	762	1	TCGA
TCGA-BR-8384	69	MALE	T4	N2	113	0	TCGA
TCGA-BR-8483	59	MALE	T3	N2	164	0	TCGA
TCGA-BR-8484	61	MALE	T4	N1	766	1	TCGA
TCGA-BR-8485	68	FEMALE	T4	N3	280	0	TCGA
TCGA-BR-8486	90	FEMALE	T1	N0	0	0	TCGA
TCGA-BR-8487	64	FEMALE	T3	N0	34	0	TCGA
TCGA-BR-8588	55	FEMALE	T4	N0	389	0	TCGA
TCGA-BR-8589	56	MALE	T4	N1	825	0	TCGA
TCGA-BR-8590	62	MALE	T4	N2	284	1	TCGA
TCGA-BR-8591	79	MALE	T4	N3	856	0	TCGA
TCGA-BR-8592	63	FEMALE	T4	N1	191	1	TCGA
TCGA-BR-8676	59	MALE	T3	N3	229	0	TCGA
TCGA-BR-8677	74	FEMALE	T3	N3	813	0	TCGA
TCGA-BR-8678	76	MALE	T2	N0	754	0	TCGA
TCGA-BR-8679	63	FEMALE	T2	N0	0	0	TCGA
TCGA-BR-8680	45	MALE	T4	N2	972	0	TCGA
TCGA-BR-8682	52	MALE	T4	N0	991	0	TCGA
TCGA-BR-8683	75	MALE	T4	N2	300	1	TCGA
TCGA-BR-8686	69	MALE	T4	N1	635	1	TCGA
TCGA-BR-8687	67	FEMALE	T4	N2	250	1	TCGA
TCGA-BR-8690	54	FEMALE	T3	N3	325	0	TCGA

TCGA-BR-A44T	53	FEMALE	T3	N0	1038	0	TCGA
TCGA-BR-A44U	70	MALE	T3	N3	422	1	TCGA
TCGA-BR-A452	57	MALE	T4	N1	229	1	TCGA
TCGA-BR-A453	51	MALE	T4	N3	185	1	TCGA
TCGA-BR-A4CQ	58	MALE	T4	N1	1064	0	TCGA
TCGA-BR-A4CR	70	FEMALE	T4	N3	0	0	TCGA
TCGA-BR-A4CS	77	MALE	T4	N3	45	1	TCGA
TCGA-BR-A4IU	34	FEMALE	T4	N1	838	0	TCGA
TCGA-BR-A4IV	47	MALE	T4	N2	869	1	TCGA
TCGA-BR-A4IY	58	MALE	T4	N0	392	0	TCGA
TCGA-BR-A4IZ	45	FEMALE	T4	N2	273	1	TCGA
TCGA-BR-A4J1	63	MALE	T4	N1	22	1	TCGA
TCGA-BR-A4J2	70	MALE	T4	N0	431	0	TCGA
TCGA-BR-A4J4	39	MALE	T4	N2	16	0	TCGA
TCGA-BR-A4J5	56	MALE	T4	N1	862	0	TCGA
TCGA-BR-A4J6	69	FEMALE	T3	N0	20	0	TCGA
TCGA-BR-A4J7	49	MALE	T4	unknow	989	0	TCGA
TCGA-BR-A4J8	71	FEMALE	T3	N3	411	0	TCGA
TCGA-BR-A4J9	55	MALE	T3	N0	14	0	TCGA
TCGA-BR-A4PD	72	FEMALE	T4	N0	628	0	TCGA
TCGA-BR-A4PE	68	FEMALE	T2	N0	621	0	TCGA
TCGA-BR-A4PF	72	MALE	T4	N2	35	0	TCGA
TCGA-BR-A4QI	70	FEMALE	T3	N0	652	1	TCGA
TCGA-BR-A4QL	75	FEMALE	T3	N3	491	1	TCGA
TCGA-BR-A4QM	65	MALE	T3	N2	156	0	TCGA
TCGA-CD-5798	82	MALE	T3	N0	408	0	TCGA
TCGA-CD-5799	45	MALE	T2	N1	396	0	TCGA
TCGA-CD-5800	51	FEMALE	T3	N0	400	0	TCGA
TCGA-CD-5801	69	MALE	T3	N1	401	1	TCGA
TCGA-CD-5802	58	MALE	T3	N0	406	0	TCGA
TCGA-CD-5803	78	FEMALE	T3	N0	341	1	TCGA
TCGA-CD-5804	90	MALE	T3	N1	0	0	TCGA
TCGA-CD-5813	60	MALE	T3	N0	377	1	TCGA
TCGA-CD-8524	61	FEMALE	T3	N0	388	0	TCGA
TCGA-CD-8525	82	FEMALE	T3	N1	383	0	TCGA
TCGA-CD-8526	73	FEMALE	T3	N1	381	0	TCGA
TCGA-CD-8527	72	FEMALE	T2	N1	218	1	TCGA
TCGA-CD-8528	43	FEMALE	T4	N0	375	0	TCGA
TCGA-CD-8529	65	MALE	T4	N0	374	0	TCGA
TCGA-CD-8530	51	MALE	T3	N0	377	0	TCGA
TCGA-CD-8531	66	FEMALE	T3	N1	383	0	TCGA
TCGA-CD-8532	52	MALE	T3	N0	354	1	TCGA
TCGA-CD-8533	48	MALE	T3	N0	468	0	TCGA
TCGA-CD-8534	41	MALE	T3	N0	367	0	TCGA

TCGA-CD-8535	59	MALE	T3	N1	390	0	TCGA
TCGA-CD-8536	74	MALE	T3	N0	378	0	TCGA
TCGA-CD-A486	68	MALE	T3	N0	192	1	TCGA
TCGA-CD-A487	51	MALE	T3	N1	374	0	TCGA
TCGA-CD-A489	58	MALE	T3	N0	344	1	TCGA
TCGA-CD-A48A	57	MALE	T3	N0	378	0	TCGA
TCGA-CD-A48C	79	FEMALE	T3	N1	353	1	TCGA
TCGA-CD-A4MG	76	MALE	T3	N0	200	1	TCGA
TCGA-CD-A4MH	86	FEMALE	T3	N0	371	0	TCGA
TCGA-CD-A4MI	62	MALE	T3	N1	358	1	TCGA
TCGA-CD-A4MJ	60	MALE	T2	N0	384	0	TCGA
TCGA-CG-4300	79	MALE	T3	N2	609	1	TCGA
TCGA-CG-4301	75	FEMALE	T4	N1	92	0	TCGA
TCGA-CG-4305	69	MALE	T2	N1	485	0	TCGA
TCGA-CG-4306	90	MALE	T3	N2	1	1	TCGA
TCGA-CG-4436	57	MALE	T2	N0	243	0	TCGA
TCGA-CG-4437	83	MALE	T2	N1	245	0	TCGA
TCGA-CG-4438	56	MALE	T4	N2	1645	0	TCGA
TCGA-CG-4440	68	FEMALE	T3	N3	122	1	TCGA
TCGA-CG-4441	83	MALE	T2	N2	426	1	TCGA
TCGA-CG-4442	85	MALE	T2	N0	0	0	TCGA
TCGA-CG-4443	68	MALE	T1	N0	912	0	TCGA
TCGA-CG-4444	76	MALE	T2	N2	1431	0	TCGA
TCGA-CG-4449	81	MALE	T2	N1	580	0	TCGA
TCGA-CG-4455	72	MALE	T2	N1	366	0	TCGA
TCGA-CG-4460	66	FEMALE	T4	N1	669	1	TCGA
TCGA-CG-4462	72	FEMALE	T3	N3	0	1	TCGA
TCGA-CG-4465	69	FEMALE	T4	N3	274	1	TCGA
TCGA-CG-4466	81	FEMALE	T2	N0	577	0	TCGA
TCGA-CG-4469	70	MALE	T3	N3	215	1	TCGA
TCGA-CG-4472	49	MALE	T4	N1	365	0	TCGA
TCGA-CG-4474	67	FEMALE	T4	N2	0	0	TCGA
TCGA-CG-4475	76	MALE	T3	N1	699	0	TCGA
TCGA-CG-4476	69	MALE	T4	N3	0	0	TCGA
TCGA-CG-4477	58	FEMALE	T2	N0	942	0	TCGA
TCGA-CG-5716	86	MALE	T4	N2	0	0	TCGA
TCGA-CG-5717	58	MALE	T2	N1	212	1	TCGA
TCGA-CG-5718	78	FEMALE	T2	N1	1095	1	TCGA
TCGA-CG-5719	54	FEMALE	T4	N0	31	0	TCGA
TCGA-CG-5720	71	MALE	T2	N0	30	1	TCGA
TCGA-CG-5721	58	MALE	T4	N1	183	0	TCGA
TCGA-CG-5722	67	FEMALE	T3	N2	30	0	TCGA
TCGA-CG-5723	83	MALE	T2	N1	2496	0	TCGA
TCGA-CG-5724	59	MALE	T3	N3	366	1	TCGA

TCGA-CG-5725	72	MALE	T2	N0	457	1	TCGA
TCGA-CG-5726	73	MALE	T1	N0	881	1	TCGA
TCGA-CG-5727	66	MALE	T3	N0	2405	0	TCGA
TCGA-CG-5728	88	FEMALE	T2	N0	579	0	TCGA
TCGA-CG-5730	80	FEMALE	T2	N1	122	0	TCGA
TCGA-CG-5732	66	MALE	T2	N3	2100	1	TCGA
TCGA-CG-5733	83	FEMALE	T2	N2	641	1	TCGA
TCGA-CG-5734	68	MALE	T3	N1	243	1	TCGA
TCGA-D7-5577	53	FEMALE	T2	N3	782	1	TCGA
TCGA-D7-5578	80	MALE	T3	N2	385	0	TCGA
TCGA-D7-5579	74	MALE	T3	N2	636	0	TCGA
TCGA-D7-6518	75	MALE	T2	N3	582	0	TCGA
TCGA-D7-6519	64	FEMALE	T2	N1	625	0	TCGA
TCGA-D7-6520	53	MALE	T2	N2	573	0	TCGA
TCGA-D7-6521	65	MALE	T2	N2	564	0	TCGA
TCGA-D7-6522	58	MALE	T2	N0	566	0	TCGA
TCGA-D7-6524	53	MALE	T2	N1	543	0	TCGA
TCGA-D7-6525	58	MALE	T2	N2	406	1	TCGA
TCGA-D7-6526	67	FEMALE	T3	N2	523	0	TCGA
TCGA-D7-6527	62	MALE	T2	N1	312	1	TCGA
TCGA-D7-6528	70	FEMALE	T2	N0	463	0	TCGA
TCGA-D7-6815	70	FEMALE	T2	N2	486	0	TCGA
TCGA-D7-6817	63	MALE	T2	N3	389	0	TCGA
TCGA-D7-6818	53	MALE	T2	N3	376	1	TCGA
TCGA-D7-6820	64	MALE	T2	N1	344	0	TCGA
TCGA-D7-6822	77	MALE	T2	N0	375	0	TCGA
TCGA-D7-8570	44	MALE	T3	N3	752	0	TCGA
TCGA-D7-8572	57	MALE	T2	N2	511	0	TCGA
TCGA-D7-8573	57	MALE	T3	N0	593	0	TCGA
TCGA-D7-8574	72	MALE	T2	N3	523	0	TCGA
TCGA-D7-8575	75	MALE	T3	N2	554	1	TCGA
TCGA-D7-8576	54	FEMALE	T3	N3	446	1	TCGA
TCGA-D7-8578	72	MALE	T2	N0	643	0	TCGA
TCGA-D7-8579	66	FEMALE	T2	N2	636	0	TCGA
TCGA-D7-A4YT	56	MALE	T2	N3	434	0	TCGA
TCGA-D7-A4YU	73	MALE	T3	N3	500	0	TCGA
TCGA-D7-A4YV	69	FEMALE	T3	N1	180	0	TCGA
TCGA-D7-A4YX	63	MALE	T3	N1	1108	0	TCGA
TCGA-D7-A4YY	61	MALE	T3	N3	419	0	TCGA
TCGA-D7-A4Z0	60	FEMALE	T2	N2	449	0	TCGA
TCGA-D7-A6ET	76	MALE	T3	N3	804	0	TCGA
TCGA-D7-A6EV	71	FEMALE	T2	N2	342	0	TCGA
TCGA-D7-A6EX	72	FEMALE	T3	N2	344	0	TCGA
TCGA-D7-A6EY	72	FEMALE	T3	N3	348	1	TCGA

TCGA-D7-A6EZ	66	MALE	T3	N2	618	1	TCGA
TCGA-D7-A6F0	79	FEMALE	T2	N0	678	0	TCGA
TCGA-D7-A6F2	62	MALE	T2	N0	476	0	TCGA
TCGA-D7-A747	57	MALE	T3	N1	255	1	TCGA
TCGA-D7-A748	41	FEMALE	T4	N3	132	1	TCGA
TCGA-D7-A74A	61	FEMALE	T3	N2	607	0	TCGA
TCGA-D7-A74B	52	FEMALE	T3	N3	217	1	TCGA
TCGA-EQ-5647	86	FEMALE	T4	N3	19	1	TCGA
TCGA-EQ-8122	71	FEMALE	T3	N1	243	1	TCGA
TCGA-EQ-A4SO	70	MALE	T4	N1	494	0	TCGA
TCGA-F1-6177	90	MALE	T1	N1	0	0	TCGA
TCGA-F1-6874	79	MALE	T2	N0	440	0	TCGA
TCGA-F1-6875	79	MALE	T2	N0	2197	1	TCGA
TCGA-F1-A448	70	MALE	T3	N3	647	0	TCGA
TCGA-F1-A72C	68	MALE	T3	N0	346	0	TCGA
TCGA-FP-7735	77	MALE	T2	N0	106	1	TCGA
TCGA-FP-7829	69	MALE	T3	N1	594	0	TCGA
TCGA-FP-7916	77	MALE	T4	N3	428	1	TCGA
TCGA-FP-7998	77	MALE	T4	N3	678	0	TCGA
TCGA-FP-8099	79	MALE	T3	N0	519	0	TCGA
TCGA-FP-8209	49	MALE	T2	N0	1811	1	TCGA
TCGA-FP-8210	48	MALE	T3	N1	153	1	TCGA
TCGA-FP-8211	62	MALE	T3	N1	413	0	TCGA
TCGA-FP-8631	68	MALE	T3	N2	17	0	TCGA
TCGA-FP-A4BE	55	MALE	T3	N0	337	0	TCGA
TCGA-FP-A4BF	68	MALE	T3	N2	168	1	TCGA
TCGA-FP-A8CX	60	MALE	T4	N3	7	0	TCGA
TCGA-FP-A9TM	77	MALE	T1	N1	189	0	TCGA
TCGA-HF-7132	unknow	MALE	T2	N1	2351	0	TCGA
TCGA-HF-7133	unknow	FEMALE	T2	N3	1918	0	TCGA
TCGA-HF-7134	unknow	MALE	T1	N0	1588	0	TCGA
TCGA-HF-A5NB	75	FEMALE	T4	N3	928	0	TCGA
TCGA-HJ-7597	71	FEMALE	T2	N0	805	1	TCGA
TCGA-HU-8238	56	MALE	T3	unknow	46	0	TCGA
TCGA-HU-8243	66	MALE	T4	N3	180	1	TCGA
TCGA-HU-8244	77	FEMALE	T1	N0	742	0	TCGA
TCGA-HU-8245	72	MALE	T3	N0	552	0	TCGA
TCGA-HU-8249	76	MALE	T3	N2	881	0	TCGA
TCGA-HU-8602	58	FEMALE	T4	N0	679	0	TCGA
TCGA-HU-8604	82	FEMALE	T3	N0	694	0	TCGA
TCGA-HU-8608	70	MALE	T4	N2	641	0	TCGA
TCGA-HU-8610	75	MALE	T1	N0	23	0	TCGA
TCGA-HU-A4G2	45	MALE	T3	N1	739	0	TCGA
TCGA-HU-A4G3	54	MALE	T2	N2	170	0	TCGA

TCGA-HU-A4G6	74	MALE	T1	N0	738	0	TCGA
TCGA-HU-A4G8	71	FEMALE	T3	N1	690	0	TCGA
TCGA-HU-A4G9	67	FEMALE	T1	N0	736	0	TCGA
TCGA-HU-A4GC	74	MALE	T4	N2	99	0	TCGA
TCGA-HU-A4GD	56	MALE	T3	N1	692	0	TCGA
TCGA-HU-A4GF	69	MALE	T3	N0	785	0	TCGA
TCGA-HU-A4GH	75	MALE	T1	N0	358	0	TCGA
TCGA-HU-A4GJ	60	FEMALE	T4	N3	650	0	TCGA
TCGA-HU-A4GN	61	MALE	T2	N1	912	0	TCGA
TCGA-HU-A4GP	62	FEMALE	T2	N1	273	0	TCGA
TCGA-HU-A4GQ	71	MALE	T4	N2	3	1	TCGA
TCGA-HU-A4GT	71	FEMALE	T3	N0	198	0	TCGA
TCGA-HU-A4GU	73	MALE	T3	N1	200	0	TCGA
TCGA-HU-A4GX	70	FEMALE	T4	N2	616	0	TCGA
TCGA-HU-A4GY	76	FEMALE	T4	N1	8	0	TCGA
TCGA-HU-A4H0	72	MALE	T4	N3	64	0	TCGA
TCGA-HU-A4H2	58	FEMALE	T3	N3	394	0	TCGA
TCGA-HU-A4H3	56	FEMALE	T4	N3	882	0	TCGA
TCGA-HU-A4H4	53	FEMALE	T2	N2	725	0	TCGA
TCGA-HU-A4H5	71	MALE	T2	N0	724	0	TCGA
TCGA-HU-A4H6	72	FEMALE	T3	N2	644	0	TCGA
TCGA-HU-A4H8	77	MALE	T1	N1	270	0	TCGA
TCGA-HU-A4HB	68	MALE	T2	N2	477	1	TCGA
TCGA-HU-A4HD	73	MALE	T3	N2	1016	0	TCGA
TCGA-IN-7806	50	MALE	T3	N1	1106	0	TCGA
TCGA-IN-7808	59	MALE	T3	N3	105	1	TCGA
TCGA-IN-8462	80	MALE	T2	N1	572	0	TCGA
TCGA-IN-8663	68	MALE	T2	N2	103	1	TCGA
TCGA-IN-A6RI	45	MALE	T1	N0	559	0	TCGA
TCGA-IN-A6RJ	64	MALE	T1	N0	379	0	TCGA
TCGA-IN-A6RL	84	MALE	T2	N1	406	1	TCGA
TCGA-IN-A6RN	72	FEMALE	T1	N2	594	0	TCGA
TCGA-IN-A6RP	63	MALE	T1	N3	196	0	TCGA
TCGA-IN-A6RR	84	MALE	T3	N1	205	1	TCGA
TCGA-IN-A6RS	76	MALE	T1	N0	383	0	TCGA
TCGA-IN-A7NR	64	FEMALE	T3	N3	198	0	TCGA
TCGA-IN-A7NT	73	FEMALE	T3	N1	323	0	TCGA
TCGA-IN-A7NU	69	MALE	T3	N3	356	0	TCGA
TCGA-IN-AB1V	63	MALE	T1	N0	479	0	TCGA
TCGA-IN-AB1X	78	FEMALE	T3	N0	411	0	TCGA
TCGA-IP-7968	74	MALE	T3	N2	77	0	TCGA
TCGA-KB-A6F5	73	MALE	T3	N2	169	0	TCGA
TCGA-KB-A6F7	54	FEMALE	T1	N1	1935	0	TCGA
TCGA-KB-A93G	68	MALE	T2	N0	613	0	TCGA

TCGA-KB-A93H	79	FEMALE	T3	N1	1145	0	TCGA
TCGA-KB-A93J	78	MALE	T2	N1	1124	0	TCGA
TCGA-MX-A5UG	78	MALE	T3	N1	113	1	TCGA
TCGA-MX-A5UJ	86	FEMALE	T3	N2	600	0	TCGA
TCGA-MX-A663	66	MALE	T3	N0	300	1	TCGA
TCGA-MX-A666	61	MALE	T2	unknow	427	0	TCGA
TCGA-R5-A7O7	51	MALE	T3	N1	1389	0	TCGA
TCGA-R5-A7ZE	66	FEMALE	T3	N2	554	1	TCGA
TCGA-R5-A7ZF	65	FEMALE	T4	N1	259	1	TCGA
TCGA-R5-A7ZI	44	FEMALE	T4	N1	2267	0	TCGA
TCGA-R5-A7ZR	70	FEMALE	T3	unknow	185	1	TCGA
TCGA-R5-A804	54	MALE	T3	N2	140	1	TCGA
TCGA-R5-A805	71	MALE	T3	N2	281	1	TCGA
TCGA-RD-A7BS	46	MALE	T3	N1	336	1	TCGA
TCGA-RD-A7BT	66	MALE	T3	N3	262	1	TCGA
TCGA-RD-A7BW	68	FEMALE	T2	N0	156	1	TCGA
TCGA-RD-A7C1	82	MALE	T2	N0	507	1	TCGA
TCGA-RD-A8MV	56	MALE	T3	N2	3720	0	TCGA
TCGA-RD-A8MW	72	MALE	T3	N2	1153	1	TCGA
TCGA-RD-A8N0	53	FEMALE	T3	N2	1236	0	TCGA
TCGA-RD-A8N1	70	MALE	T3	N2	3519	0	TCGA
TCGA-RD-A8N2	59	FEMALE	T2	N0	3540	0	TCGA
TCGA-RD-A8N4	58	FEMALE	T3	N1	2171	0	TCGA
TCGA-RD-A8N5	78	MALE	T3	N1	1747	1	TCGA
TCGA-RD-A8N6	78	FEMALE	T2	N2	272	1	TCGA
TCGA-RD-A8N9	63	FEMALE	T2	N1	1083	0	TCGA
TCGA-RD-A8NB	80	FEMALE	T3	N1	513	1	TCGA
TCGA-SW-A7EA	61	FEMALE	T2	N0	579	0	TCGA
TCGA-SW-A7EB	45	MALE	T3	N2	176	0	TCGA
TCGA-VQ-A8DL	69	FEMALE	T3	N0	28	1	TCGA
TCGA-VQ-A8DT	43	MALE	T3	N3	1484	0	TCGA
TCGA-VQ-A8DU	63	MALE	T3	N2	166	1	TCGA
TCGA-VQ-A8DV	48	MALE	T2	N0	403	1	TCGA
TCGA-VQ-A8DZ	70	MALE	T4	N3	396	1	TCGA
TCGA-VQ-A8E0	68	MALE	T3	N2	562	1	TCGA
TCGA-VQ-A8E2	57	MALE	T3	N2	1319	0	TCGA
TCGA-VQ-A8E3	79	MALE	T3	N0	661	1	TCGA
TCGA-VQ-A8E7	59	MALE	T3	N1	1138	0	TCGA
TCGA-VQ-A8P2	68	MALE	T4	N1	1160	0	TCGA
TCGA-VQ-A8P3	72	MALE	T4	N1	1132	0	TCGA
TCGA-VQ-A8P5	67	MALE	T3	N0	235	1	TCGA
TCGA-VQ-A8P8	72	FEMALE	T4	N0	942	0	TCGA
TCGA-VQ-A8PB	65	FEMALE	T3	N0	1043	1	TCGA
TCGA-VQ-A8PC	65	MALE	T3	N1	1407	1	TCGA

TCGA-VQ-A8PD	69	MALE	T4	N3	496	1	TCGA
TCGA-VQ-A8PE	78	MALE	T3	N3	675	1	TCGA
TCGA-VQ-A8PF	76	MALE	T3	N3	76	1	TCGA
TCGA-VQ-A8PH	62	MALE	T3	N3	389	1	TCGA
TCGA-VQ-A8PJ	53	MALE	T4	N2	82	1	TCGA
TCGA-VQ-A8PK	58	MALE	T3	N3	543	1	TCGA
TCGA-VQ-A8PM	56	MALE	T4	N3	57	1	TCGA
TCGA-VQ-A8PO	74	MALE	T4	N0	282	1	TCGA
TCGA-VQ-A8PP	76	MALE	T4	N1	712	1	TCGA
TCGA-VQ-A8PQ	50	FEMALE	T4	N1	476	1	TCGA
TCGA-VQ-A8PS	76	MALE	T3	N1	406	1	TCGA
TCGA-VQ-A8PT	65	MALE	T4	N0	900	0	TCGA
TCGA-VQ-A8PU	72	FEMALE	T4	N1	832	1	TCGA
TCGA-VQ-A8PX	51	MALE	T1	N0	1964	0	TCGA
TCGA-VQ-A8PY	47	FEMALE	T3	N2	436	1	TCGA
TCGA-VQ-A8PZ	56	FEMALE	T3	N0	2233	0	TCGA
TCGA-VQ-A91A	67	MALE	T3	N3	1200	0	TCGA
TCGA-VQ-A91D	70	MALE	T4	N2	356	1	TCGA
TCGA-VQ-A91E	67	FEMALE	T4	N0	664	0	TCGA
TCGA-VQ-A91K	69	MALE	T3	N1	1862	0	TCGA
TCGA-VQ-A91N	59	FEMALE	T4	N3	570	1	TCGA
TCGA-VQ-A91Q	61	MALE	T3	N3	633	1	TCGA
TCGA-VQ-A91S	63	MALE	T4	N1	1000	0	TCGA
TCGA-VQ-A91U	78	MALE	T3	N1	52	1	TCGA
TCGA-VQ-A91V	58	MALE	T3	N2	1297	0	TCGA
TCGA-VQ-A91W	30	MALE	T3	N1	1851	0	TCGA
TCGA-VQ-A91X	74	MALE	T3	N2	289	1	TCGA
TCGA-VQ-A91Y	67	MALE	T4	N3	296	1	TCGA
TCGA-VQ-A91Z	67	FEMALE	T3	N1	1690	0	TCGA
TCGA-VQ-A922	70	MALE	T4	N1	275	1	TCGA
TCGA-VQ-A923	90	MALE	T3	N1	0	1	TCGA
TCGA-VQ-A924	69	MALE	T3	N0	1686	1	TCGA
TCGA-VQ-A925	66	MALE	T3	N2	138	1	TCGA
TCGA-VQ-A927	81	MALE	T3	N3	200	1	TCGA
TCGA-VQ-A928	49	MALE	T3	N3	174	1	TCGA
TCGA-VQ-A92D	67	MALE	T2	N0	2032	0	TCGA
TCGA-VQ-A94O	74	MALE	T4	N3	640	1	TCGA
TCGA-VQ-A94P	57	MALE	T4	unknow	81	1	TCGA
TCGA-VQ-A94R	63	MALE	T4	N2	1294	1	TCGA
TCGA-VQ-A94T	73	MALE	T3	N2	342	1	TCGA
TCGA-VQ-A94U	70	MALE	T4	N0	819	0	TCGA
TCGA-VQ-AA64	68	MALE	T3	N2	560	1	TCGA
TCGA-VQ-AA68	52	FEMALE	T4	N3	1328	0	TCGA
TCGA-VQ-AA69	35	MALE	T3	N2	864	0	TCGA

TCGA-VQ-AA6A	56	MALE	T4	N3	1184	0	TCGA
TCGA-VQ-AA6B	48	MALE	T3	N2	1002	0	TCGA
TCGA-VQ-AA6D	52	FEMALE	T4	N1	521	0	TCGA
TCGA-VQ-AA6F	57	MALE	T3	N1	1646	0	TCGA
TCGA-VQ-AA6G	68	MALE	T3	N0	792	1	TCGA
TCGA-VQ-AA6I	68	MALE	T3	N3	491	1	TCGA
TCGA-VQ-AA6J	75	MALE	T4	N2	838	0	TCGA
TCGA-VQ-AA6K	59	MALE	T4	N3	378	1	TCGA
TCGA-ZA-A8F6	71	MALE	T2	N0	525	0	TCGA
TCGA-ZQ-A9CR	79	FEMALE	T4	N3	24	1	TCGA

Table S3: Univariate Cox regression analysis and Kaplan–Meier analysis of 91 AAM genes associated with OS in GC patients

Gene	HR	HR.95L	HR.95H	pvalue	km
ADI1	1.00174646397098	0.858118587898907	1.1694141022343	0.982368851655377	0.327261274863479
ALDH18A1	0.799712349528558	0.674710662806953	0.947872736037478	0.00995767210312522	0.0028404816083667
ALDH4A1	0.9719597370333	0.834059744240584	1.13265954499938	0.715625061699372	0.100446981316063
ALDH5A1	0.836274426383648	0.747090555188134	0.936104614583418	0.00188651702777313	0.000416559574462738
ALDH6A1	0.991062396651671	0.835868823165565	1.17507035414623	0.917708368238673	0.0631509025289497
AMT	0.944557720924208	0.837825001720617	1.06488740050162	0.351166158653554	0.0258622698042629
ARG1	1.42983696883856	1.02935506403356	1.98613076176676	0.032964163291279	0.0202950589517812
ASL	1.00082365612439	0.863212020737436	1.16037307937682	0.991295879436772	0.096904617856806
ASMTL	1.11985135472322	0.945882061001551	1.32581757111194	0.188820328169196	0.00665898919465457
ASPA	1.12868168760335	0.929703860312815	1.37024530747081	0.221200690666622	0.0136266557409527
ASRGL1	0.964332265440422	0.844111970629395	1.10167460067658	0.592914729544902	0.0409067796289762
ATF4	0.898894273141465	0.711759789330325	1.13522978735109	0.37079405912389	0.103273453290716
BAAT	1.03341637629638	0.95570852463128	1.11744258764414	0.409865079176275	0.166309429265701
BBOX1	1.06273930288991	0.875681882353449	1.28975470278266	0.537879211440503	0.181748769256794
BCAT1	1.05284443491271	0.953597113351494	1.16242109860298	0.308017808268028	0.0100512736549896

BCKDHA 0.903658613966065 0.744884372003528 1.09627604133867
0.304148079876206 0.036665976937089
BCKDHB 0.876415332096402 0.731271007705279 1.05036823043751
0.153290168409827 0.0129485811877016
BCKDK 1.10859381307574 0.88847167535153 1.38325202309184
0.361315104038893 0.0223563855177843
BPHL 0.988564561357313 0.834644088745518 1.17087020102234
0.894043263438464 0.068935225436323
CDO1 1.25749393806685 1.12329660047948 1.40772348425153
6.91380819554865e-05 5.50210532157536e-05
COLQ 1.13849597747286 0.890515906617442 1.45553053133583
0.300740050290794 0.0694668183649071
DCT 1.36630300284808 0.63250048809065 2.95143471150039
0.427047030892985 0.0316487568495282
DDAH1 0.864266858280976 0.764766791227345 0.976712392445944
0.0194106309681599 0.00520172855819467
DDAH2 1.14327401370393 0.998065289170769 1.30960918548392
0.0533569740932295 9.41054522721751e-05
DDO 1.06246262139361 0.882944510672644 1.27847991375882
0.521117919984694 0.049553230046522
DHPS 0.923561022545136 0.71434604126805 1.19405010049542
0.544022744878366 0.0188708212736672
DIO1 1.20641085628642 1.01756474724529 1.43030422202205
0.0307391248526856 0.0443657947961452
DIO2 1.11992317618927 1.01929994537434 1.23047972901171
0.0183768040427819 0.00377396722434864
ETNK1 0.90983385480201 0.795845213344879 1.040149050925
0.166484493221839 0.0129708999219375
FAH 1.252048809374 1.0711959121544 1.46343558938909 0.00474250906988168
0.000660120646866491
FARS2 1.14875821134392 0.905303685610694 1.4576825976798
0.253759860268709 0.00481266737780217
FPGS 0.92060035262286 0.728540781736865 1.16329110256375
0.488318753964278 0.00025197574203073
GAD1 0.81982051860333 0.736465329923223 0.912610078729845
0.00028173374820204 0.000958238277350754
GAD2 1.451922503893 0.920207755413119 2.29087284356185
0.109027145811798 0.0805120219676523
GAMT 1.05321958510304 0.960628975762248 1.15473457748287
0.269410135753702 0.0356167154234883
GATM 1.02478091632845 0.953931819942761 1.10089201818846
0.503057374081466 0.0735196558226684
GCLC 0.953667616874221 0.800296257925679 1.13643155827303
0.595897468115777 0.173803326893341

GCLM	1.15181962433143	0.973180407147603	1.36325026403227	0.100218087004504	0.0428118361484553
GCSH	0.977667177621329	0.804518896388809	1.18808037261578	0.82034492083209	0.0460430815137004
GGT1	0.96336395171671	0.86745409490799	1.06987805915617	0.485443429746908	0.00778135333840169
GGT5	1.22058746150676	1.0850364790562	1.37307250027523	0.000904068720004667	4.98633577727947e-06
GLDC	0.971985116251252	0.898251654033474	1.05177102872193	0.480223918784569	0.0676942117183205
GLS2	0.571256507883707	0.442996654196104	0.736651156861848	1.58991431827653e-05	9.22527976232601e-09
GLUD1	0.92360768917798	0.76226863320075	1.11909519341855	0.417211325467283	0.0495523610545477
GLUD2	1.0638826489952	0.858433006683366	1.31850276261631	0.571636635352258	0.211829504059333
GOT1	0.736858847460897	0.617131728922253	0.87981371826339	0.000737276152581613	6.3079321200199e-05
GOT2	0.825919893942639	0.681684837652132	1.00067308752189	0.0508085966776446	0.00555708806906674
GSS	0.888858515100246	0.746624324857068	1.05818874842775	0.18541972031342	0.0136893855710744
GSTZ1	0.792834194076367	0.647317507880444	0.971062966232669	0.0248462744999556	0.0129265844741671
HDC	1.0813914674453	0.979785039635591	1.19353476380741	0.120111411515459	0.0150463709539435
HGD	1.04374351918494	0.957486623790451	1.13777102130984	0.330639080158697	0.0574359354993736
HPD	1.16184700494843	0.971661232639785	1.38925833156923	0.100018853987814	0.000135467488746155
HPRT1	0.928503141017326	0.77985218748765	1.10548908717742	0.404650425788632	0.0262600771276817
MARS2	0.751839315988572	0.626449090455626	0.902327684209806	0.00218385881882696	0.000161471513919098
MAT1A	1.01946234650283	0.889628525532191	1.16824432457956	0.78153065363508	0.160692058280003
MAT2B	0.883348583793041	0.725291346645473	1.07585003474554	0.217531679841845	0.0279656657884771
MCCC2	0.888283051986453	0.756174930502154	1.04347122420785	0.149303638836523	0.00833293866524931
MSRA	0.966251609375152	0.844127314613349	1.10604426187503	0.618498000676938	0.0810515303577689
MTHFR	0.774206042260862	0.647090535273415	0.926292324179899	0.00516316234074466	0.000941858441157239

NFS1	0.756247001058717	0.638144530973139	0.896206891780721	0.00126041506877505	0.000103304450621233
OAZ1	0.847231128548354	0.628373665924973	1.14231487426308	0.276905575045745	0.0174622047105574
OTUB2	0.956804999301164	0.809056388100743	1.13153523061202	0.605878009843561	0.0242287569942863
P4HB	1.09450293763531	0.919530139203443	1.30277043613825	0.309614310840122	0.0659715246773801
PAH	1.01895530362876	0.862340710242213	1.20401356269213	0.825452147803527	0.00379635747601759
PEPD	1.20055968371531	1.00704849097877	1.43125536364365	0.0415212641056748	0.0159555848018774
PLOD1	1.16208859617085	0.980314578004041	1.3775679110066	0.0834733771652625	0.0252859868291549
PRG3	1.11384197201441	0.644012608751838	1.92642802603733	0.699708799241656	0.0211082062719685
PTS	1.01356930406881	0.813775914163853	1.26241477078624	0.904227962965163	0.0771674166000599
PYCR1	0.99111948397238	0.87164683189233	1.12696770706673	0.891735530690999	0.153156643414112
QDPR	0.823011217104925	0.680181448362126	0.995833486949077	0.0451884560068993	0.00765673361105756
SARS2	0.903810704938695	0.74900672650851	1.09060941838217	0.291384712520831	0.0103293012786352
SCLY	0.609496333366849	0.473027149042266	0.785337123122379	0.000128981983105709	0.000715864600808858
SDS	1.0104080667276	0.908799231630868	1.12337733767241	0.848150049064186	0.125812833533185
SLC25A15	0.761596041797367	0.655975033278384	0.884223486346107	0.000349778489485684	0.00102849904335112
SLC3A1	1.01137484719326	0.941911355901881	1.08596108872239	0.755380567296961	0.115560582353611
SLC5A7	1.21823651443969	0.926891865377868	1.60115787024318	0.156903622325038	0.00182061284675883
SLC6A14	0.970913931617269	0.908099851745129	1.03807291763888	0.387047551812105	0.0709696221494099
SLC6A6	1.06036556310239	0.899292950984454	1.25028793585293	0.485639661757652	0.0499452720331957
SLC7A2	1.15279911317564	1.06688611154273	1.24563042011754	0.000320156932551117	2.60307587429676e-06
SLC7A4	1.02316782616606	0.93041887588225	1.12516246997753	0.636635380231267	0.135735265454388
SLC7A5	0.986085933061717	0.904153584406813	1.07544280546113	0.751552906431935	0.0673805410979365

SLC7A6	0.819373091435239	0.689246332764805	0.974067225392457	0.0239617476645511	0.00032347241040831
SLC7A7	1.00418993187887	0.886642606077796	1.13732118485452	0.947516447631222	0.0102821166883966
SLC7A8	0.926776722584183	0.820516785709604	1.0467977114948	0.221001444290149	0.0168755160267675
SLC7A9	0.998669874130724	0.929352205571036	1.07315774527426	0.971071525253462	0.00991778616183858
SMS	0.953779868817382	0.800235873884654	1.13678487537092	0.597213734442098	0.0202726898671511
SNCAIP	1.06528440042038	0.985255087672938	1.15181425397087	0.112476373523116	0.000942817151252662
SULT1B1	0.969501081987901	0.894766297936978	1.05047804118558	0.449187959818148	0.033811632046732
TGFB2	1.27789496188588	1.1423749258349	1.42949175151043	1.80974008142564e-05	1.86341887198349e-06
TYR	2.50909620519858	1.32737835151791	4.74285553907269	0.0046295745132407	1.07303122397484e-05
YOD1	0.944245460977272	0.815421954980489	1.09342100139739	0.443334811615382	0.18595490964165

Table S4: Univariate Cox regression analysis of 51 AAM genes associated with OS in GC patients

Gene	HR	HR.95L	HR.95H	pvalue
ABCA8	1.17882522730858	1.08485972039834	1.28092958970666	0.000103696663481678
C14orf132	1.18011942485332	1.08772988686242	1.28035634005916	6.84191570938418e-05
MFAP4	1.10462498705652	1.03768800093171	1.17587980292152	0.00180905263181363
SETBP1	1.1339809558188	1.03389065011499	1.24376094127237	0.00765569863961746
OGN	1.0954012441409	1.03826834731432	1.15567799863034	0.000855906380018473
RGMA	1.12116570143558	1.05528916213135	1.19115459078227	0.000214079043842086
FHL1	1.12422718905317	1.06074747771842	1.1915058005369	7.85925376397373e-05
SFRP1	1.09279669071199	1.03527359267519	1.15351595528019	0.00129788834513371
PODN	1.14685302297636	1.06995568561618	1.22927694482278	0.000109077928077847
RERG	1.18100922176595	1.09038421840114	1.27916633270923	4.42322813697123e-05

MGP	1.14864117160047	1.07926762182168	1.22247393919662	1.30097383906572e-05
SPARCL1	1.16019620377598	1.08277016799795	1.24315877093757	2.47950526517536e-05
C7	1.10080906898222	1.04847645355646	1.15575376275081	0.000111165726996023
SVEP1	1.16151444826887	1.07178797730792	1.25875251645013	0.000262138799070512
FBLN1	1.11222089269384	1.04216006329529	1.18699167019815	0.00135556233203594
CYBRD1	1.16456351410663	1.0746674177551	1.26197943287551	0.000201724259591929
PGM5	1.09875052922391	1.03883615476985	1.1621204363428	0.000995671546506279
FRZB	1.12495353719087	1.04565133546742	1.21027001822988	0.00159496220523995
RCAN2	1.12779767057667	1.03852578423986	1.22474338630805	0.00425754130409298
HSPB8	1.14173414764464	1.07304044418054	1.21482547183328	2.83115752151946e-05
PBK	0.871959358025581	0.795318489219901	0.955985724403499	0.0035126628711943
CHRDL2	1.12001747270527	1.05712604659836	1.18665048808669	0.000121005182346884
CH25H	1.14766062518601	1.05201146787862	1.25200622884685	0.00192246135881624
SPON1	1.12541000760327	1.0567010680212	1.19858654783547	0.000237022814455758
PPP1R14A	1.18878804676826	1.10672868890217	1.27693176684609	2.15008296869133e-06
PTGIS	1.07753002789566	1.00898141274845	1.150735728475	0.0259753876693945
THBS4	1.0895934476232	1.04198145061962	1.13938101335434	0.000167267765135649
RAI2	1.17182827487594	1.05902644144892	1.29664515639478	0.00213693875083002
APOD	1.12070233178318	1.06854368592275	1.17540698898019	2.7804716676723e-06
CPE	1.15229796341795	1.06895546114065	1.24213837223893	0.000214933427918499
RBPMS2	1.1446063745883	1.07855388310704	1.21470403404793	8.44829838258775e-06
EFEMP1	1.1382095521346	1.05777947825036	1.22475526440853	0.000535666836759358

PALM	1.14623424705007	1.04526555765193	1.25695612898781	0.00372030261126714
PPP1R3C	1.13992238949547	1.06485923325882	1.22027683424072	0.000164461556107565
PSAT1	0.893872858172021	0.822822943092409	0.971057860362655	0.00793140964814335
ZSCAN18	1.13285479536731	1.03552710354082	1.23933017590604	0.00649546340482605
MYH11	1.09662699007269	1.04934223868804	1.1460424549949	4.1010364840195e-05
PTGER1	1.11907203302804	1.02839912802108	1.21773947583495	0.00906681658809494
CGNL1	1.10194085880887	1.00991426359558	1.2023532096569	0.0291326630661333
PDK4	1.15629396836594	1.08215755102125	1.23550932118682	1.74392222652968e-05
RGS5	1.18522148598597	1.08286866540931	1.29724870218847	0.000226311803251064
CBX6	1.11422287163255	1.01696423330989	1.22078296070298	0.0202897388698102
KIT	1.13846378038783	1.04739762864079	1.23744769303793	0.00229888135923155
SGCE	1.27323445506077	1.16154651453583	1.39566169521994	2.51008385594641e-07
DES	1.07860741124751	1.04176940944061	1.11674804141422	1.97275466534454e-05
ACTG2	1.10373951592336	1.05400002415152	1.15582627238688	2.72389341986843e-05
MATN2	1.10558262391399	1.02570931378293	1.19167577195192	0.00870478069594842
REEP1	1.11769653632824	1.03372240054092	1.20849228638796	0.00523426307044846
MYOM1	1.15152032928628	1.05893945904317	1.25219535209096	0.000969827147252959
CPA3	1.10192043228343	1.02930965756953	1.17965340182547	0.00526132107831538
AGMAT	0.877865576026485	0.799459508606213	0.963961227899922	0.00635477904324659
